# Evolution of Cancer Vaccines—Challenges, Achievements, and Future Directions

**DOI:** 10.3390/vaccines9050535

**Published:** 2021-05-20

**Authors:** Ban Qi Tay, Quentin Wright, Rahul Ladwa, Christopher Perry, Graham Leggatt, Fiona Simpson, James W. Wells, Benedict J. Panizza, Ian H. Frazer, Jazmina L. G. Cruz

**Affiliations:** 1 Faculty of Medicine, Diamantina Institute, University of Queensland, Brisbane, QLD 4102, Australia; b.tay@uq.net.au (B.Q.T.); q.wright@uq.edu.au (Q.W.); g.leggatt@uq.edu.au (G.L.); f.simpson@uq.edu.au (F.S.); j.wells3@uq.edu.au (J.W.W.); i.frazer@uq.edu.au (I.H.F.); 2 Department of Medical Oncology, Princess Alexandra Hospital, Brisbane, QLD 4102, Australia; rahul.ladwa@health.qld.gov.au; 3 Faculty of Medicine, University of Queensland, Woolloongabba, QLD 4102, Australia; c.perry@uq.edu.au (C.P.); ben.panizza@health.qld.gov.au (B.J.P.); 4 Department of Otolaryngology, Princess Alexandra Hospital, Brisbane, QLD 4102, Australia

**Keywords:** vaccine, cancer, antigens, ICIs, immunotherapies, T-cells, tumour, neoantigens

## Abstract

The development of cancer vaccines has been intensively pursued over the past 50 years with modest success. However, recent advancements in the fields of genetics, molecular biology, biochemistry, and immunology have renewed interest in these immunotherapies and allowed the development of promising cancer vaccine candidates. Numerous clinical trials testing the response evoked by tumour antigens, differing in origin and nature, have shed light on the desirable target characteristics capable of inducing strong tumour-specific non-toxic responses with increased potential to bring clinical benefit to patients. Novel delivery methods, ranging from a patient’s autologous dendritic cells to liposome nanoparticles, have exponentially increased the abundance and exposure of the antigenic payloads. Furthermore, growing knowledge of the mechanisms by which tumours evade the immune response has led to new approaches to reverse these roadblocks and to re-invigorate previously suppressed anti-tumour surveillance. The use of new drugs in combination with antigen-based therapies is highly targeted and may represent the future of cancer vaccines. In this review, we address the main antigens and delivery methods used to develop cancer vaccines, their clinical outcomes, and the new directions that the vaccine immunotherapy field is taking.

## 1. Introduction

We are witnessing an inflection point in the field of vaccine development. The benefits that will flow for human health and the global economy following the recent release of Covid-19 vaccines are undeniable. The successful development of the first documented vaccine against smallpox in 1796 encouraged the discovery and adoption of new vaccines to prevent diseases caused by known and emerging pathogens [1]. However, in the more than 220 years since vaccination against smallpox became routine, which has resulted in the elimination of the disease globally, the USA Food and Drug Administration (FDA) has approved fewer than 100 vaccines for infectious diseases, highlighting the many successes but also reminding us of the challenges that remain [2].

The concept of vaccination has now expanded beyond interventions that prevent disease, and towards approaches that target disease-specific antigens to treat or ameliorate ongoing pathology. These therapeutic vaccines stem from the realization that in addition to eliciting new immune responses in naïve individuals, vaccines are capable of enhancing pre-existing immunity and modulate its type to better tackle the targeted disease (e.g., systemic vs. mucosal; Th1 vs. Th2) [3,4]. Antigen specific immunization has the potential to alter not only the course of acute and chronic infectious illness, but autoimmunity, graft rejection, and cancer [5,6,7]. However, in contrast to the success of prophylactic vaccines against hepatitis B virus (HBV) and human papillomavirus (HPV) in preventing liver and cervical cancer, most of the clinically tested cancer therapeutic vaccines have shown at best a modest efficacy. One of the reasons for this is that many immunogens trialled in these vaccines have been non-mutated self-antigens to which natural tolerance has been induced and therefore only weak anti-tumour responses are achieved. Conversely, viral antigens and mutated self (neo) antigens that are not subject to thymus-induced tolerance can now be identified through genomics and proteomics practices, offering a diverse range of personalized tumour specific-antigens with the potential to overcome the problems of innate or tumour induced tolerance [8].

In addition to the comprehensive definition of relevant antigens, improvements in vaccine delivery technologies, including more powerful adjuvants and novel antigen expression systems, have returned antigen-based therapies to the spotlight [9]. Importantly, the past 10 years of cancer treatment and management has dramatically improved with the discovery and adoption of immune checkpoint inhibitor monoclonal antibodies (ICIs), which in combination with tumour-antigen specific vaccines are being trialled to treat some of the most devastating cancer types, and are currently showing promising results [10].

We are living in exciting times in the fields of immunology and cancer immunotherapy. This article will comprehensively review the array of tumour-specific antigens and delivery methods, and the most relevant clinical trials (Table 1) summarizing what we have learned to date to show how cancer vaccines are becoming a powerful tool to treat cancer in the future.

## 2. Cancer Vaccine Antigens

Tumour antigens can be classified into two general classes: (i) tumour-associated antigens (TAAs) that are expressed by the tumour as well as the healthy tissue and (ii) neoantigens, for which expression is restricted to the tumour lesion.

### 2.1. Tumour Associated Antigens

TAAs are self-proteins differentially expressed in malignant cells as a result of genetic amplification and/or post-translational modification [11]. TAAs can be subdivided into three distinct types: (1) overexpressed antigens (2) differentiation and lineage-specific antigens and (3) cancer-germline/cancer testis antigens [12]. One TAA, prostatic acid phosphatase (PAP), is the protein antigen overexpressed in prostatic cancer, that is the basis of one of the few therapeutic cancer vaccines approved for clinical use, Sipuleucel-T [13]. This section of the review will examine other TAAs, and their clinical potential that these have.

#### 2.1.1. Overexpressed Antigens

Overexpressed TAAs are defined as self-proteins present at increased levels within tumour cells in comparison to normal cells and tissues. Overexpressed TAAs encompass a larger and more diverse group of antigens than the other subcategories.

A sponsored study by the National Cancer Institute (NCI, USA), based on the features and clinical results of 75 TAAs, was conducted with the aim to tabulate the relevance of pre-defined and pre-weighted criteria for vaccine development and ascribed the following characteristics to TAAs in in order of importance: (i) therapeutic function, (ii) immunogenicity, (iii) mechanism of action in oncogenicity, (iv) specificity to a cancer type, (v) expression rates, (vi) stem cell expression, (vii) patient number expressing the TAA of interest, (viii) number of antigenic isotopes and lastly, (ix) the cellular location of antigen expression [8]. Based on these criteria, overexpressed TAAs accounted for 40% of the final list (30/75). Overexpressed TAAs commonly used in clinical studies have included mucin 1 (MUC1), Human Epidermal Growth Factor Receptor-2 (HER2/neu), Tumour Protein 53 (p53), Human Telomerase Reverse Transcriptase (hTERT), and survivin.

MUC1, or mucin 1, is a highly glycosylated transmembrane protein expressed on the glandular or luminal epithelial cells of the duodenum, oesophagus, lungs, mammary glands, pancreas, prostate, stomach, and uterus. On healthy tissues, MUC1 oligomerizes through negatively charged sugar residues, resulting in a gel like layer that protects the underlying epithelial tissue against damage caused by pH changes, pollutants, pathogenic microbes, and cell desiccation [74].

MUC1 is overexpressed by many human epithelial cancers, in particular breast cancer [75]. Aberrant glycosylation of MUC1 results in random additions of sugar chains to MUC1′s five O-glycosylation sites on cancer cells. The expression of these aberrant forms of MUC1 facilitates novel combinations between cell surface receptors and ligand interactions, which can potentially contribute towards the tumour cell survival [76]. The overexpression of MUC1 can also partly be due to a loss of cell polarity, which leads to MUC1 expression not only on the cell surface, but also in the cytoplasm [74,76]. Additionally, MUC1 can undergo modifications in tertiary structure through the formation of new carbohydrate sub-chains (Thomsen-Friedenreich (TF/T) and sialyl-Tn (STn)), which can lead to exposure of the protein core peptide, that is also recognized as a TAA associated with carcinoma differentiation and invasion [77]. MUC1 can also be altered by up and down-regulation of its functional enzymatic partners ST6 β-galactoside α-2,6-sialyltransferase 1 (ST6GAL1) and 2 β 1,6-N-acetylglucosaminyltransferase-1 (C2GnT-1), involved in the biosynthesis of mucin type O-glycan [78].

MUC1′s physiological properties and widespread expression in many different types of human epithelial carcinomas has led it to become the primary focus of many cancer vaccine development projects. However, clinical trial results involving MUC1-based cancer vaccines to date have had limited success. Three vaccines have progressed to Phase III clinical trials. These include the peptide vaccine Tecemotide (L-BLP25), the glycopeptide vaccine STn-KLH, and the viral vaccine PANVAC-V/F [79]. Tecemotide was tested in a Phase III clinical trial in patients with non-small cell lung cancer (NSCLC) after chemotherapy and did not increase patients’ overall survival (OS), with median OS reported between tecemotide and placebo at 25.6 months and 22.3 months, respectively. Interestingly, in the subgroup of subjects who had received concurrent chemoradiotherapy prior to trial entry, the vaccinated subjects experienced an extended life expectancy of 10.2 months compared to patients who received placebo (30.8 months to 20.6 months). However, no benefit from Tecemotide over placebo was seen in the subgroup of subjects who had received prior sequential chemoradiotherapy, with median OS reported at 19.4 month and 24.6 months between Tecemotide and placebo [14].

A glycopeptide vaccine STn-KLH based on conjugated MUC1 glycopeptides and glycoforms of Tn and S-Tn induced antibody responses in mice but failed to improve OS for breast cancer patients [15].

PANVAC-V/F is another MUC1 vaccine comprising of recombinant vaccinia and fowlpox viruses expressing MUC1 and CEA (Carcinoembryonic antigen). CEA is a protein postulated to be involved in promoting cell proliferation, with overexpression observed mostly within adenocarcinomas of the gastrointestinal tract and pancreas. PANVAC-V/F was trialled in stage IV pancreatic cancer after Phase I and II clinical trials demonstrated favourable safety and early efficacy results [16,17]. However, in early 2006, it was announced by Therion Biologics that Phase III trials showed no significant improvement in OS of pancreatic cancer patients in comparison to supportive care and the trial, alongside with its Biologics License Application (BLA) was terminated although results of the Phase III clinical trial for PANVAC-V/F remain unpublished [18].

HER2/neu is one of a family of four transmembrane tyrosine kinases (Epidermal Growth Factor (EGFR), Human Epidermal Growth Factor Receptor-2, -3, and -4 (HER2, HER3 and HER4)). HER2 is part of a transmembrane signalling system that consists of at least twelve ligands and four receptors. Through the binding of its extracellular domain, HER proteins undergo dimerization and transphosphorylation, where phosphorylated tyrosine residues interact with intracellular signalling molecules to activate downstream pathways that results in a multitude of biological effects, including cell proliferation, cell survival, differentiation and angiogenesis. As HER2 possesses the strongest catalytic kinase activity, paired with its open conformation setting, it is the most common dimerization partner within its family of four [80]. The overexpression and aberrant expression of HER2/neu is seen in some ovarian, prostate, gastric, and lung cancers [81], but it is most prevalent in breast cancer, with ~30% of tumours overexpressing HER2 [82]. Many trials of immunotherapy have targeted HER2/neu, with significant impact of HER2-directed monoclonal antibodies (Mab), including trastuzumab, pertuzumab, neratinib, lapatinib, and T-DM1 in HER2-positive breast cancer patients [83]. The success of Mab therapy such as trastuzumab and pertuzumab against HER-2 associated cancers has encouraged clinical trials of immunotherapeutic immunization based on the HER2/neu antigen. Many Phase I/II clinical trials are in progress [84], and one peptide-based vaccine (E75) reached Phase III trials [85]. E75 is a Her2 derived 9-amino-acid peptide from the extracellular domain of HER2 and is predicted to be strongly presented by some Human Leucocyte Antigen Class-I alleles (HLA) including HLA-A*02 and HLA-A*03. Early open label Phase I/II clinical studies trialled the combination of E75 and Granulocyte-Macrophage Colony-Stimulating Factor (GM-CSF), an immunostimulatory adjuvant commonly utilized to elicit antitumoral effects [86]. The treatment was administered in patients with recurrent or persistent refractory HER2+ breast cancer suggested a 10% increase in disease-free survival (DFS) between a cohort of HLA A*02/A*03 patients given E75 and GM-CSF and the negative control group consisting of patients of other HLA-types who received no treatment [19]. However, a Phase III trial by the same group demonstrated that the previous combination therapy did not increase the DFS of HER2+ breast cancer patients when compared with GM-CSF alone [20].

The protein p53, or tumour protein 53, is a 53 kilodalton (kDa) protein that is encoded by the TP53 tumour suppressor gene. P53 interacts with a plethora of gene regulatory sequences and proteins to control cell cycle, apoptosis and genetic stability, by activating DNA repair proteins thereby inducing growth arrest at the G1/S point where cells can be checked for damage, and by initiating programmed cell death when cell damage is irreversible through its direct interaction with pro-apoptotic genes [87]. This protein works within a network, activated when cells are damaged or under stress following DNA damage, or in response to chemotherapeutic drugs, ultraviolet light, or protein-kinase inhibitors. Thus, TP53 is classified as a tumour suppressor gene. With its widespread effects as a tumour suppressor, the TP53 gene is one of the most commonly mutated genes in human cancer [88]. Most mutations to the TP53 gene are missense and cause single amino-acid changes at many different positions. These mutations alter the p53 protein structure, along with its functional and transcriptional activity. Mutated, dysfunctional p53 can result in the inhibition of downstream cell regulatory effects and contributes to the progression of malignant cell growth [87,89]. Mutations in this gene are associated with cancers of the adrenal gland, bladder, breast, brain, head and neck (H&N), liver, small cell lung cancer (SCLC), colorectal, bone, muscle, and ovary, which makes p53 an ideal antigen for cancer vaccine design [89].

Key clinical studies of p53 vaccines, including the synthetic long peptide (SLP)-p53^®^, a viral canary pox virus-based vaccine that encodes for the wild-type human p53 (ALVAC) and a novel live attenuated Modified Vaccinia Ankara (MVA) virus-based vaccine (MVAp53), have shown strong induction of vaccine-specific cellular responses with modest clinical outcomes [90]. Two independent Phase II clinical trials of the SLP-p53^®^ vaccine or its combination with cyclophosphamide in patients with recurrent ovarian cancer suggested that, regardless of the intensity of the detected vaccine-induced cellular response, two out of 20 and two out of 10 patients respectively achieved stable disease by the end of the trial [21,22]. Similarly, a Phase I/II trial of SLP-P53^®^ with metastatic colorectal cancer patients showed the generation of vaccine-specific T-cells in nine out of 10 patients, lasting up to 6 months in 6 of the individuals [23]. SLP-p53^®^ has also been tested as part of the combination therapy with PegIntron (IFN-α) alongside gemcitabine chemotherapy in patients with platinum-resistant ovarian cancer, reporting strong specific vaccine-induced p53-specific T-cell responses [24]. Later studies trialled ALVAC as a therapeutic vaccine in a phase I/II dose escalation study with advanced colorectal cancer patients reporting strong p53-specific IFN-γ-producing T-cell immunity in two out of five patients that received the highest dose [25].

Most recently, a phase I trial of a MVA vaccine expressing wild-type p53 (MVAp53) administered in combination with gemcitabine chemotherapy in platinum-resistant ovarian cancers showed that ~50% of patients had increased levels of p53-reactive CD4^+^ and CD8^+^ T-cells which significantly correlated with a longer progression-free survival (PFS) of 7.0 months in comparison to vaccinated patients with no-detected cellular response (2.3 months) [26].

Telomeres are nucleoproteins consisting of 5–20 kb of repeating hexanucleotides of the DNA sequence ‘TTAGGG’ associated with the Shelterin protein complex. Located at the end of eukaryotic chromosomes, they are also known as Telomeric DNA, and regulate and maintain genomic stability and structural integrity. As a result of end replication, telomers are shortened throughout a cell’s lifespan. Other factors such as oxidative damage, age and epigenetics may contribute to the shortening of telomers [91]. To counteract the end replication problem, Shelterin recruits a reverse transcriptase known as telomerase, with the ability to elongate the 3′ overhang through the addition of telomeric repeats [92]. The telomerase enzyme consists of a large ribonucleoprotein complex composed by two subunits, the human telomerase RNA component (hTERC or hTR) and human telomerase reverse transcriptase (hTERT) encoded by the TERT gene [93,94]. In normal cells, the shortening of telomers is usually progressive and associated with minimal telomerase activity. As hTERT is not commonly expressed in normal cells, it is used as a cancer biomarker for diagnosis and prognosis [91]. However, the regulation of hTERT has also been shown to be associated with cancer progression. Amplifications of the TERT gene can occur as result of telomere dysfunction in addition to the formation of chromosomal fusions. Prior studies demonstrate that 3% out of 95% of hTERT expressing tumours were associated with hTERT amplification [95]. Besides amplifications of the TERT gene, mutations in the form of genomic rearrangements to the hTERT gene locus (5p15.33) and the shift in the proximity between the active enhancers to the hTERT gene promoter region, result in increased expression [96]. The TERT promoter region can also be susceptible to genetic alterations and methylation events that can lead to expression of mutant or methylated hTERT wherein many instances, associate with elevated expression of the hTERT protein in tumours [95]. Within the cancer, the activated synthesis of telomers results in the immortalization and uncontrolled proliferation of malignant cells. Its widespread effects, observed in over 90% of tumour types, along with its significant immunogenicity make hTERT an ideal candidate for cancer vaccine design [97].

Over the years, hTERT antigen-based vaccines’ clinical progress has had limited success, but nonetheless there have been many significant studies and today, hTERT vaccine developments are still consistently pursued. To date, a single-phase III clinical trial of a peptide vaccine (GV1001), consisting of 16 amino-acids derived from hTERT’s active site in combination with GM-CSF as the adjuvant, was capable of inducing CD4^+^ and CD8^+^ T-cell responses in patients with advanced metastatic pancreatic cancer. However, whilst the vaccine was generally well tolerated and induced cellular immune responses, the study showed that GV1001 peptide vaccine did not improve disease outcome [27]. Currently, GV1001 is being tested in a multitude of clinical trials, for a range of different indications including hepatocellular carcinoma, melanoma, NSCLC, colon, and pancreatic cancers [98].

Recent efforts in the development of a hTERT antigen-based vaccine include the peptide-based vaccines UVI and VX-001. The early Phase I/IIa of the UVI + GM-CSF vaccine with metastatic hormone naïve prostate cancer on androgen deprivation therapy and conformal radiotherapy resulted in 17 of 21 patients displaying clinically stable disease, of which 18 patients developed vaccine-specific immunity and 14 patients PSA’s (Prostate-Specific Antigen) levels decreased to <0.5 ng/m. Vaccination conferred long-lasting protection, with 17 patients clinically stable at nine months post-treatment [28].

More modest results were achieved using the hTERT peptide vaccine VX-001. Recently, a randomized double-blind phase IIb trials in patients with metastatic TERT^+^ NSCLC showed that post chemotherapy, vaccination (OS 11.3 months) did not significantly extend the OS compared to the placebo group (14.3 months). However, solace can be taken as the patients who developed a vaccine-specific immune response experienced a significant increase in OS (21.3 compared to 13.4 months for non-responders) and extended time to treatment failure (9.1 compared to 3.6 months non-responders) [29].

Survivin is a member out of 8 other proteins in the inhibitor of apoptosis (IAP) protein family. As survivin lacks a functional caspase activation and recruitment domain (CARD) motif, it is speculated that it cooperates with other members of the IAP family to inhibit apoptosis [99]. Survivin is also showed to have a role in the regulation of cell division as a key component of the chromosomal passenger complex (CPC), which aids in the proper segregation of chromosomes [100]. Reduction in survivin concentrations have been shown to contribute towards mitotic failures and increased apoptosis. As a result, due to mainly transcriptional depression and/or altered splicing, overexpression of survivin in commonly associated with cancer progression [99,101]. Furthermore, through undetermined mechanisms, survivin is shown to upregulate Vascular Endothelial Growth Factor (VEGF) and its pro-angiogenic role is responsible for survivin mediated tumour progression [102]. Since its discovery, there have been many strategies employed to include survivin in the formulation of new anti-cancer therapeutics, leading to the development of several survivin inhibitors. To date, direct inhibitors of survivin, along with inhibitors that disrupt its homodimerization, that decrease its transcription and those that induce its mRNA degradation have been extensively investigated [103]. Conversely, successful survivin vaccines have been less abundant, with developments to date including SurVaxM, to treat malignant glioblastoma [30], and the multi-epitope vaccine EMD640744 to treat solid tumours in a wide variety of indications [103].

SurVaxM (SVN53-67/M57-KLH) peptide vaccine to treat glioma contains the 53–67 amino-acid survivin sequence with a mutation in position 57 to enhance antigenicity conjugated to the protein Keyhole Limpet Hemocyanin [30]. Phase I trials with HLA-A*02 or HLA-A*03 survivin^+^ glioma patients treated with SurVaxM in incomplete Freund’s adjuvant (Montanide ISA-51) and GM-CSF (Sargramostim) reported vaccine-specific immune responses in 6 out of 8 participants. A median PFS and OS of 17.6 and 86.6 weeks respectively was reported, representing a significant advancement with respects to historical chemotherapy data (PFS of 10 weeks and OS of 30 weeks). Preliminary results of an active SurVaxM in Montanide ISA-51 with Sargramostim Phase II clinical trials with resected newly diagnosed glioblastoma patients (nGBM) on temozolomide chemoradiation indicated that 86% of the patients reached the one-year OS from initial vaccination, with a median PFS of 13.9 months from diagnosis. Furthermore, contributing to the general one-year OS mentioned, O6-Methylguanine-DNA Methyltransferase (MGMT) promoter methylation status correlated with overall survival, where the OS at 12 months for patients with observable methylation of MGMT (meMGMT) was 93.1% in contrast to unmethylated MGMT (unMGMT) OS-12 of 78% [31]. The latest updates as of 1 February 2021, have been reported by the trials coordinator mainly including the observed adverse events (NCT02455557). Based on these promising results, in March 2020 the recruitment of recurrent glioblastoma patients started to assess the clinical activity of SurVaxM in combination with Pembrolizumab, an anti-programmed cell death-1 (anti-PD-1) checkpoint inhibitor in a Phase II study (NCT04013672).

EMD640744 is a combination of five survivin peptides presented by HLA-A*01, A*02, A*03, A*024, or B*07 alleles assessed in Phase I clinical trials with the Montanide ISA-51 adjuvant in patients who expressed at least one of the mentioned alleles with advance solid tumours: colorectal, ovarian, lung kidney, rectum, breast, testicle cancers, as well as melanoma and mesothelioma [32]. Although no dose-dependent effect was observed, 63% of vaccinated patients developed anti-survivin T-cell responses. Unfortunately, despite promising results, there has not been further developments of EMD640744.

Whilst overexpressed proteins, such as MUC1, HER2/neu, p53, hTERT, and survivin, have been prominent TAAs targets in the development of cancer immunotherapies, only modest potential efficacy have been achieved over the last two decades. Despite the plethora of clinical trials, there are no approved vaccines with overexpressed TAAs. Prior review of overexpressed antigens and their utility in cancer vaccine design suggests that the concept is impeded by high immunological tolerance that limits vaccine’s ability to reach the activation threshold for T-cell recognition [104]. Additionally, as many of these antigens are prevalent within healthy cells and tissues, strong vaccine responses could contribute to the induction of autoimmunity. Further, varied tumour specificity for some overexpressed antigens, adds to the uncertainty of prospects of targeting overexpressed antigens which appear to be less ideal than other TAAs types and classes of tumour antigens.

#### 2.1.2. Normal Differentiation Antigens

The use of normal differentiation antigens as targets for tumour immunotherapy, has a storied past with the completion of multiple Phase III clinical trials within recent years, along with the development of a first-in-class vaccine approved by the FDA to treat prostate cancer (Sipuleucel-T (Provenge)) [13].

Differentiation antigens are a type of TAA that are only expressed during certain stages of differentiation in the normal tissue. Therefore, these antigens are restricted to the tumour and its corresponding tissue of origin which makes targeting differentiation antigens less likely to lead to off-target effects. Following up from the sponsored study conducted by Cheever et al., the list of the 75 most prominent tumour associated antigens includes 20 different types of differentiation antigens (20/75) [8]. To date, key developments of cancer vaccines expressing differentiation antigens include gp100-based vaccines against metastatic melanoma and Sipuleucel-T (Provenge), a prostatic acid phosphatase-based cancer vaccine which is currently in use to treat prostate cancer patients.

Gp100, or more commonly known as Melanocyte protein PMEL, is a glycoprotein with a size of 100 kDa encoded by the PMEL gene. The gp100 protein is initially synthesized within the endoplasmic reticulum as an integral membrane glycoprotein. However, through post-translational modifications, proteolytic processing and precise oligomerization events, the end product is a fibrillar structure [105]. These fibrillar sheets are laterally assembled and are an essential component for melanosome maturation [106]. As membrane bound organelles, these fibrillar sheets help melanosomes store and polymerize synthesized melanin [107]. Individual fibril units are also shown to possess biophysical properties similar to that of amyloids, therefore gp100 belongs to a class of proteins known as functional amyloids [106]. As it is integral in the development of skin melanocyte, gp100 was identified to be a melanocyte differentiation antigen by Bakker et al. in 1994, where the team postulated the potential of the melanocyte lineage-specific antigen to serve as a key target against melanoma [108].

Two Phase III clinical trials have been completed for gp100 peptide vaccines against metastatic melanoma. In 2010, a Phase III study by Hodi et al. assessed the clinical efficacy of the gp100 vaccine composed of two modified HLA-A*02:01-restricted peptides (gp100:209–217 and gp100:280–288) in combination with ipilimumab, an anti-cytotoxic T-lymphocyte-associated protein 4 (CTLA-4) ICI in HLA-A*02:01^+^ unresectable stage II/IV melanoma patients [33]. Although vaccinated patients produced vaccine-specific T-cell responses, the trials revealed that improved median OS at two years between the treated groups was associated to ipilimumab treatment (OS-24 months 23.5% ipilimumab vs. 13.7% placebo) and no clinical advantage was achieved with the co-administration of the gp100 vaccine (OS-24 months 21.6% ipilimumab + gp100 vaccine).

The Phase III study conducted by Schwartzentruber et al., investigated the clinical response and toxicity of the gp100:209–217 (210M) peptide vaccine with Montanide ISA-51 adjuvant +/− Interleukin-2 (IL-2) with HLA-A*02:01 patients with stage IV or locally advanced stage III cutaneous melanoma [34]. It was reported that the combination therapy with IL-2 + gp100:209–217 induced higher response rate (16% combination vs 6% IL-2 alone) and longer median PFS than the treatment with IL-2 alone (PFS = 2.2 months combination vs. 1.6 months IL-2 only, *p* = 0.008), although the clinical efficacy of the gp100:209–217 (210M) vaccine as a monotherapy was not tested.

##### PAP, PSA and the Sipuleucel-T (Provenge) Vaccine

Human prostatic acid phosphatase (PAcP or PAP) is another 100 kDa glycoprotein identified as a prostate epithelium-specific differentiation antigen. PAP is part of a small group of at least five acid phosphatases (AcPs) with the ability to hydrolyse a large variety of small organic phosphomonoesters within acidic environments [109]. It mainly consists of two primary subunits both with sizes of approximately 50 kDa each [110]. The PAP protein can be detected in two forms, cellular (cPAP) or secretory (sPAP), differentiated by post-transcriptional modifications [109]. Cellular PAP is primarily localized in the columnar epithelial cells of prostate but can also be expressed by many non-prostatic cells such as the kidney, lungs and placenta to name a few [111]. In normal differentiated prostate epithelia of adults, cPAP is found at concentrations of 0.5 mg/g wet prostate tissue, whereas sPAP is found in seminal fluids at 1 mg/mL [112,113]. However, in prostate cancer, it is shown that cPAP concentrations decrease in comparison to normal adjacent tissue. It is postulated that cPAP levels correlates inversely to progression rates for prostate cancer. On the other hand, sPAP is showed to increase along with cancer progression [114,115]. Hence, prior to the adoption of the gold standard, with prostate-specific antigen (PSA) as a diagnostic indicator, PAP levels were used as a prostate cancer marker. PAP has ultimately been used as antigen in the design of the first successful therapeutic cancer vaccine [116].

Early proof-of-concept studies using human peripheral blood dendritic cells (DCs) pulsed with different HLA-A*02:01-restricted PAP were able to induce PAP-specific cytotoxic T-cells ex vivo [117]. Furthermore, in preclinical studies, it was shown that immunization with DCs loaded with PAP fused to GM-CSF was able to overcome tolerance and induced the production of PAP-specific antibodies in rat models [118]. This and other preclinical studies marked the start of a long journey to the development of Sipuleucel-T. Phase I/II studies with Sipuleucel-T (Provenge^®^), autologous antigen presenting cells (APCs) loaded ex vivo with a recombinant fusion protein consisting of PAP linked to GM-CSF (PA2024), with hormone-refractory prostate cancer resulted in all patients developing immune responses to PA2024, of which 38% had a specific response against PAP [35]. Levels of prostate-specific antigen (PSA) decreased by >50% in three patients, signs of less cancer-induced autoimmune prostatitis, and between 25% and 49% in another three patients. The former results led to the first Phase III trials with 127 asymptomatic metastatic hormone refractory prostate cancer patients enrolled, 115 of which were afflicted by progressive disease [36]. Median PFS and OS for vaccinated patients were moderately increased from 10 to 11.7 weeks and from 21.4 to 25.9 months in comparison to the placebo cohort, respectively. In parallel (2006), Higano and peers conducted two simultaneous Phase III trials (D9901 and D9902A) the integrated results from which indicated a 33% reduction in risk of death in Sipuleucel-T vaccinated patients with median PFS increasing from 9.7 to 11.1 weeks and median OS from 18.9 to 23.2 in comparison with placebo group [37]. A 3-years follow-up showed an improvement of 4.1 months in OS for patients treated with Sipuleucel-T. The final multicentre Phase III study leading to the FDA approval of Sipuleucel-T recruited 512 patients, 341 of which were vaccinated with Sipuleucel-T [38]. Similar to Higano et al. clinical trials, a 22% reduction in risk of death and increased OS from 21.7 to 25.8 in vaccinated patients compared to the placebo group were reported. The three-year follow-up study showed an increase in survival probability due to vaccination from 23% to 31.7% compared to the control group. With sufficient data to demonstrate Sipuleucel-T’s benefits, Provenge^®^ was approved by the FDA in 2010 for the treatment of minimally symptomatic/asymptomatic patients with metastatic castrate-resistant prostate cancer. Since then, the use of this vaccine has been investigated in other indications of prostate cancer, with recent Phase III trials with newly diagnosed prostate cancer patients (NCT03686683).

Despite challenges faced throughout the development of a normal differentiation antigen vaccines, Sipuleucel-T demonstrates that the underlying principles of a TAA-based vaccine are achievable. However, similar to overexpressed antigens, differentiation antigens are impeded by high central tolerance, alongside with suboptimal tumour specificity. As such, targeting differentiation antigens are less ideal than targeting the last type of tumour associated antigens, namely cancer-germline/cancer testis antigens.

#### 2.1.3. Cancer-Germline/Cancer Testis Antigens

Cancer testis antigens, also known as cancer-germline antigens (CGAs) are the third the last type of TAAs. Unlike differentiation and overexpressed antigens, cancer-germline antigens are only expressed in human tumours and germline tissues [119]. Furthermore, factors such as the blood-testis barrier and the lack of expression of HLA-I molecules on germ cell surfaces, cumulatively results in the formation of an immune privileged zone where cancer-germline antigens can avoid immunological responses [120]. The expression in various forms of cancer and their critical role in disease initiation and progression, make cancer-germline antigens attractive targets for cancer vaccine development. Referring to the study conducted by Cheever et al., the compiled list of 75 “pivotal” tumour associated antigens for research include 12 different cancer-testis antigens [8]. Between this list of twelve, critical clinical developments have been made with Melanoma-Associated Antigen 3 (MAGE-A3) and New York Esophageal Squamous Cell Carcinoma 1 (NY-ESO-1) antigen vaccines.

MAGE-A3 (MAGE3), or melanoma antigen family A3, is part of a protein subfamily of 11 proteins (MAGE-A) within the MAGE family of >40 known human proteins sharing a MAGE homology domain, a centralized and conserved 165-171 amino-acid module [121]. The MAGE-A, B and C subfamilies are classified as cancer testis antigens (CTAs) in humans, clustered on the X chromosome [122]. Collectively, the MAGE proteins have been shown to be broadly expressed in a wide variety of cancer tumour types, such as colon, brain, lungs and skin [123]. MAGE proteins bind to specific E3 ring ubiquitin ligases via the MAGE homology domain. MAGE proteins are associated with the ubiquitination of proteins through this binding interaction. By modulating the activity of cognate E3 ligases, these proteins can (1) enhance ligase activity, (2) induce highly specific ubiquitination of the E3 ligase complex and (3) alter the subcellular localization of E3 ligases. Hence, malignant expression of MAGE can contribute towards tumorigenesis through its cellular interactions with ubiquitin [123,124]. Referring to MAGE-A3 specifically, it has been shown to act in a complex with Transcription intermediary factor 1-beta (TRIM28), resulting in the ubiquitination of the alpha catalytic subunit of AMP-activated protein kinase (AMPK) and its subsequent degradation. As AMPK is a tumour suppressor, its reduction in protein levels in tumours links MAGE-A3 with tumour progression [123,125]. In 1994, van der Bruggen et al., proved that HLA-A*02 and HLA-A*01-restricted MAGE-3 peptides from two different *in vitro* patient-derived melanoma cell lines were recognized by cytotoxic T-lymphocytes (CTLs) [126,127]. Since the discovery of MAGE-A3 in 1991, there have been two independent Phase III clinical trials: MAGRIT (2016) with NSCLC patients, and DERMA (2018) with melanoma patients. MAGRIT multicentre trials recruited a total of 2312 completely resected stage IB, II, and IIIA MAGE-A3+ NSCLC patients to test the clinical efficacy of the recombinant MAGE-A3 protein (recMAGE-A3) supplemented with an AS15 immunostimulant (3-O-desacyl-4′-monophosphoryl lipid A (monophosphoryl lipid A; MPL), QS-21 (extract from the soap bark tree [Quillaja saponaria]) + a synthetic oligodeoxynucleotide containing unmethylated CG dinucleotides (CpG ODNs 7909), in a liposomal formulation) [39]. Whilst the vaccine was well tolerated with infrequent treatment-related adverse events, the trial was terminated due to a lack of efficacy observed (DFS of 60.5 vs. 57.9 months in vaccinated vs. placebo group).

Similarly, the DERMA multicentre trials enrolled 895 patients with MAGEA3+ stage III melanoma and reported no differences in DFS between MAGE-A3 + AS15 vaccinated patients (11.0 months) and placebo controls (11.2 months) [40].

NY-ESO-1 is an antigen commonly expressed in myxoid or round cell liposarcoma [128]. NY-ESO-1 is encoded by the gene CTAG1, located on the Xq28 region of the X chromosome. It is a 18 kDa polypeptide that includes a Pcc-1 domain (Transcription factor Pcc1) [129]. It is a TAAs restricted to germ and placental cell, detected during embryonic development as early as 13–18 weeks, with peak concentrations detected at 22–24 weeks [130]. RNA expression of NY-ESO-1 has also been detected in ovarian and endometrial tissues, however its functions and mechanism are unknown [131]. Whilst the function of NY-ESO-1 is not clear, it is suggested that through its Pcc-1 domain the protein is playing a role in cell regulation and growth [132]. NY-ESO-1 is also showed to be co-expressed with MAGE-C1, implicating that it might be involved in MAGE associated cellular functions [133]. Lastly, the restricted expression of the protein indicates a role in germ cell renewal or differentiation. The expression of NY-ESO-1 has been reported across a range of cancers, with examples including synovial sarcoma, oesophageal, ovarian and prostate cancers. NY-ESO-1 is expressed by various cancer types, and predominantly by 89–100% of myxoid and round cell liposarcoma [129].

Proof of concept studies in a single melanoma patient revealed pre-existing humoral and cytotoxic CD8^+^ T-cell responses to NY-ESO-1 expressed by the patient’s tumour, clearly suggesting the NY-ESO-1′s innate potential to stimulate antitumour responses [134]. Early work demonstrated the presence of immunogenic peptides within NY-ESO-1 able to be presented in HLA-A*02 and HLA-DRB1*0401 alleles to activate both CD8^+^ and CD4^+^ T-cells, respectively [135]. Recently, the focus has shifted back to cancer germline antigen with a Phase I study conducted by Ishihara et al. where a novel polysaccharide-based antigen delivery system known as cholesteryl pullulan (CHP) was used with NY-ESO-1 antigen (CHP-NY-ESO-1 vaccine) plus a the adjuvant (MIS416, a non-toxic microparticle that activates the immune system via the nucleotide-binding oligomerization domain 2 (NOD2) and TLR9 pathways) was given to NY-ESO-1-expressing refractory solid tumour patients (prostate cancer, urothelial cancer and synovial sarcoma) [41]. Despite inducing anti-NY-ESO-1 antibodies in 21 out of 26 patients, neither increase in vaccine-derived T-cell immunity nor beneficial clinical responses upon vaccination was observed.

In parallel, the Phase II clinical trials conducted by Cebon et al. of NY-ESO-1 18-mer peptides plus ISCOMATRIX microparticle delivery system (cholesterol, phospholipid and saponin) with resected stage IIc, IIIb, IIIc and IV NY-ESO1+ melanoma patients, reported that, regardless the strong cellular NY-ESO-1-specific immunity generated by the vaccine, no differences in survival or relapse end-points between the vaccinated and only-adjuvant patients (DFS = 4.67 months combination vs 5.79 months only adjuvant) were achieved [42].

Despite current challenges, it is believed that NY-ESO-1 is a promising antigen of choice, given its cellular functions and its predisposed role in cancer. This is further supported by the fact that, the National Institutes of Health (U.S) (NIH) has currently reported ~50 NY-ESO-1 associated vaccines undergoing clinical trials (active, recruiting, and proposed).

### 2.2. Neoantigens

Neoantigens are encoded by genes containing non-synonymous mutations in tumour cells, which result in unique amino-acid changes with the potential to be targeted by the immune system [136]. One of the first studies to notice the capacity of the immune system to recognize and mount a response against neoantigens is from the 1950s. Prehn et al., observed that murine sarcomas induced by methylcholanthrene treatment although histologically similar displayed different antigenicity between animals [137]. At the time, the authors concluded that this differential effect was induced by “antigens that were peculiar to and specific for the tumour tissue”- fast forward, today we know and identify them as neoantigens. Thirty years later, Boon et al. officially identified the first neoantigen by describing a new surface antigen generated as a result of a point mutation (tum-variant-P91 in the position 137-base-pair exon) in the murine P815 tumour cells [138]. Transfecting the murine P815 cell line with DNA encoding the protein variant tum-variant-P91, they confirmed that the transfected tumours were recognized by cytotoxic T-lymphocytes in the absence of detectable antibodies and rejected by syngeneic mice in contrast to the parental cell lines. Ever since, numerous studies have identified the relevance of cancer neoantigens in mounting an anti-tumour cellular response.

#### 2.2.1. Tumour-Specific Antigens

Neoantigen are part of a new class known as tumour-specific antigens due to their “non-self” characteristics. The collective total number of mutational events occurring within malignant tumour cells are known as tumour mutational burden. The three most common forms of tumour associated mutations include point mutations, frameshift mutations and also insertion/deletion events [139]. Tumours that are characterized by a higher tumour mutational burden show more abundant neoantigen generation, offering an unique target opportunity [140]. In comparison to TAAs, neoantigens offer very attractive characteristics: (i) they possess higher binding affinities to HLA and T-cell receptors, (ii) their expression is restricted to tumour cells, and (iii) the collective effect of specificity and binding affinities allow neoantigens to bypass central tolerance and issues associated with autoimmunity. Although binding affinity of neoantigens to HLA complexes and their recognition by T-cell receptors are varied, literature agrees that binding affinity to HLA correlates positively with elevated T-cell responses [136]. Supported by the ability to evade tolerance and autoimmunity, the clinical prospects of neoantigens in vaccine development are theoretically promising. Unfortunately, only ~1.2% of endogenous neoantigens are believed to possess antitumoral effects, where the larger population of neoantigens are not recognized spontaneously by T-cells [141]. Hence, the development of neoantigen-based cancer vaccine is determined by the principal basis of predicting, identifying, and validating neoantigens able to elicit the desired anti-tumour response.

#### 2.2.2. The Framework behind the Development of Neoantigen-Based Cancer Vaccines

**Figure 1:**
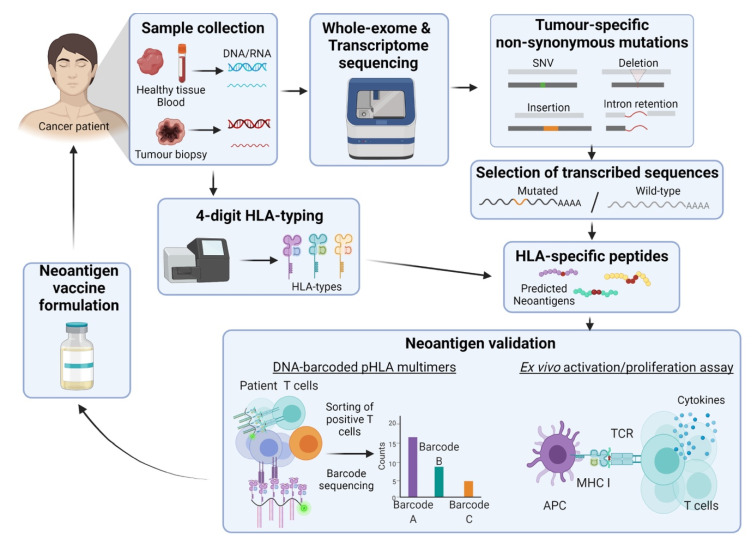
**Basic neoantigen vaccine pipeline. 1. Sample collection.** Tumour biopsy and healthy tissue from blood or the surrounding area of the lesion are the starting material for DNA and RNA extraction. **2. Whole exome and transcriptome sequencing.** DNA and RNA are used to sequence the whole-exome and transcriptome of the healthy and tumour samples. **3. Tumour-specific non-synonymous mutations.** Exome data is processed, and tumour non-synonymous mutations derived from different mutational sources are annotated using specialized packages, such as GATK, VarScan2, FACTERA and ANNOVAR [148,149,150,151]. Comparison of these mutations with the corresponding healthy tissue sequence will pinpoint somatic mutations restricted to the tumour. **4. Selection of transcribed sequences.** One of the most important quality control points is the verification that the identified exome mutations have been translated to mRNA and the evaluation of the abundance of the mutated mRNA respect to the wild-type variant. **5. 4-digit HLA-typing. Neoantigens are HLA-type specific.** Therefore, patient’s individual HLA-typing are commonly performed using DNA extracted from blood with commercial protocols such as Illumina TruSight HLA v2 Sequencing Panel^®^. **6. HLA-specific peptides.** Long peptides (~19 mer) containing the mutated regions are the input of software such as netMHCpan and netMHCIIpan that predict HLA binding of 8-10-mer sequences for each patient’s HLA-type [152]. **7. Neoantigen validation.** Some neoantigen pipelines include the use of software to predict neoantigen immunogenicity based on parameters that include strength of binding to their specific HLA or recognition by the TCR (i.e., NAseek [153], Luksza’s algorithm [154]). Additional wet-lab work can be done to ease the selection of the most promising candidates. For example, DNA barcoded MHC-I multimers can be used to detect neoantigen-specific CD8^+^ T cells in clinical samples [146]. Unique DNA barcodes (up to 1000) are bound to peptide-loaded-HLA molecules (pHLA) and joined to a fluorescently labelled backbone to generate HLA multimers. Patient’s samples are incubated with a mix of HLA multimers, and HLA multimer^+^ T cells are sorted based on the fluorescent label. Then, DNA barcodes are sequenced, and the relative number of DNA barcode counts is used to determine the composition of neaontigen-specific T cells in the patient’s sample. Ex vivo stimulation of patient’s cells with APC loaded with the neoantigens of interest, is a common validation protocol [155]. T cell proliferation and cytokine release are two of the major readouts of this method. Often, the immunogenicity of the neoantigen is compare with the one exhibited by the wild-type sequence. **8. Neoantigen vaccine formulation.** Patient-specific neoantigen vaccine will be formulated with the selected candidates using the most convenient adjuvants and delivery platforms. (Created in BioRender.com).

Neoantigens are perceived to be key to unique, personalized cancer vaccines. In the past, the comprehensive and fast identification of neoantigens along with progress into the field of research was limited by technologies. Today, advancements in the field of high-throughput screening, including whole-genome and whole-exome sequencing, are contributing to the identification of neoantigens closing the gap between theory and practice. These pipelines include the sequencing and comparison of healthy and cancer tissues to identify tumour-specific non-synonymous mutations (Figure 1) [142]. Bioinformatic algorithms are integral to this pipeline by using different parameters that include translation rate of mutated vs. wild-type sequences to protein/peptide, probability of immunological tolerance, protein/peptide recognition by APCs, HLA binding affinity and binding affinity between the peptide-HLA and the T-cell receptors. To date, there are more than a dozen algorithm-based software with one of the newest programs being NeoPredPipe developed by Schenck et al. in 2019 [143]. Based on what is observed by sequencing, and predictive algorithms, key neoantigens are selected for antigenicity verification and presentation using mass spectrometry and affinity chromatography [142,144]. As only a small proportion of predicted neoantigens exist, validation is a crucial step in the selection of these tumour antigens incentivizing the development of new technologies for this purpose [145]. One of the latest approaches utilizes DNA barcodes to label Major Histocompatibility Complex (MHC) loaded with the neoantigens of interest to test the presence of T-cell clones able to recognize these structures in clinical samples [146,147].

Once a neoantigen candidate has been identified, the selection of an appropriate administration strategy is also critical for the vaccine design. Currently, the development of neoantigen vaccines is mostly centred in peptide, DCs and DNA/RNA formulations. Between the three listed, each possesses its own features and associated benefits. However, there are no predetermined indicators as to which option is the best, whereby the method is determined on a case-by-case basis, normally influenced by the neoantigen’s nature. The vaccine delivery vectors, along with other delivery formats will be further discussed in following sections.

#### 2.2.3. Clinical Progress on Neoantigens

The highly personalized approach in targeting cancer has witnessed some clinical developments over the last few years. Currently, there are >50 clinical trials associated to neoantigen derived vaccines and immunotherapies that are in plan, recruiting or active registered in the NIH database. Clinical trials using neoantigens have historically targeted a wide variety of indications, with a non-exhaustive list that includes melanoma, breast, ovarian, prostate and lung cancer [156,157]. Most if not all of these prospective vaccines are currently within early stages of the clinical development pipeline, but some published studies highlight the potential of neoantigen vaccines. In 2015, Carreno et al. released the first results of a patient derived autologous DC vaccine loaded with unique combinations of seven HLA-A*02:01-restricted neo-peptides obtained from three Stage II resected cutaneous melanoma patients’ whole-exome sequencing data and validated by mass spectrometry [158]. The selected peptides belong to two different categories: (i) dominant T-cell immunity was detected prior to vaccination and (ii) subdominant T-cell immunity was achieved through vaccination. The three treated patients showed an increased neoantigen specific T-cell response for both dominant and subdominant neoantigens in addition to an increased T-cell TCRβ repertoire when pre- and post- vaccination clones were compared.

Following the pursuits of Carreno et al., two Phase I studies were published simultaneously in 2017 by Ott et al. and Sahin et al., respectively. The Phase I study by Ott et al. of 20 predicted patient-specific neo-peptides plus TLR3, melanoma differentiation-associated protein 5 (MDA-5) and modified polyinosinic-polycytidylic acid (poly-ICLC) immunostimulants, with six stage IIIB/C and IVM1a/b untreated high-risk melanoma patients, resulted in all patients developing neoantigen-specific CD4^+^ and CD8^+^ polyfunctional T-cell responses, which were shown to distinguish between mutated and wild-type antigens [43]. Four of the six patients showed no disease recurrence at 25 months post-vaccination. The remaining two received anti-PD-1 ICI therapy, resulting in total regression. On the other hand, Sahin et al.’s Phase I trial tested patient-unique RNA-based poly-neoantigen vaccine consisting of ten different mutated sequences delivered by two synthetic RNA chains containing five linker-connected 25-mer peptides each, given to 13 patients with stage III and IV melanoma [44]. All patients developed T-cell-specific response to at least three of the mutations, with enhancement of pre-existing and *de novo* responses. The majority of the neoantigen-specific immunity came from the CD4^+^ T-cells compartment, although two patients showed ex vivo neoantigen-specific killing of resected autologous tumour cells. Eight out of 13 patents remained relapse-free for the duration of the follow-up period (12–23 months) and two out of five patients with recurrent disease showed objective response to vaccination with delayed relapse. A third patient of this group experience complete response with vaccine complementary anti-PD-1 therapy.

Outside the melanoma realm, in 2019, both Keskin et al. and Hilf et al. demonstrated the potential of neoantigen-based cancer vaccines against glioblastoma in two separate Phase I clinical trials. Firstly, Keskin et al.’s Phase I/Ib study of 12 (20-mer) predicted patient-specific neoantigens with poly-ICLC was given post-radiotherapy to 8 newly diagnosed meMGMT patients [45]. Unfortunately, five patients discontinued therapy due to disease progression and only neoantigen-specific T-cell response able to migrate from peripheral blood to the brain were detected in the two patients who did not receive the drug dexamethasone, a potent corticosteroid prescribed to treat cerebral oedema. Overall, the median PFS and OS for all eight treated patients were 7.6 months and 16.8 months, respectively.

Hilf et al.’s Phase I trial investigated a two-step system, the Glioma Actively Personalized Vaccine Consortium (GAPVAC), consisting of 7 non-mutated TAA peptide vaccine (APVAC1) followed by six non-mutated tumour-HLA class I peptides part of each patient immunopeptidome + 14 (19-mer) predicted HLA class I-binding neoantigens vaccine (APVAC2) with poly-ICLC and GM-CSF as adjuvants in newly diagnosed HLA-A*02:1 or HLA-A*24:2 glioblastoma patients during TMZ maintenance therapy [46]. The “off-self” APVAC1 vaccine was personally formulated based on each patient’s ranking of peptides from a list of pre-defined non-mutated HLA-Class I TAAs defined by the data collected from 30 glioblastoma samples. Fifteen patients received APVAC1, of which 11 patients received the following APVAC2. APVAC1 induced CD8^+^ T-cell-specific responses against at least one peptide in 12 out of 13 patients, resulting in total of 45 immunogenic antigens of 87 tested. Eight out of 10 patients vaccinated with APVAC2 developed predominantly CD4^+^ T-cell neoepitope-specific immunity, with a total of 11 out of 13 mutated peptides being immunogenic. In contrast, APVAC2 non-mutated peptides led to no detectable immunological response. All vaccinated patients (*n* = 15) showed a median OS of 29.0 months from diagnosis and a median PFS of 14.2 months.

### 2.3. Oncogenic Viral Antigens

Approximately 15% of all human cancers are driven by viruses [159]. Currently, there are three major types of viruses clearly associated with cancer development: retroviruses, DNA and non-retroviral RNA viruses; each with their own proposed tumorigenic mechanism of action. To date, since the discovery of the Epstein-Barr Virus’s association with cancer, other viruses identified includes the hepatitis B/C virus (HBV/HCV), the human T-lymphotropic virus-1 (HTLV-1), the human papillomavirus (HPV), the Kaposi sarcoma herpesvirus (KSHV/HHV8) and the Merkel cell Polyomavirus (MCV). Due to their high immunogenic and tumour-specific properties viral antigens are ideal candidates for cancer vaccine design [160]. To date, successful virus-based prophylactic cancer vaccines include the Hepatitis B Engerix-B, Pediarix, Recombivax HB, and Twinrix vaccines and the HPV Cervarix, Gardasil, and Gardasil 9 vaccines. Whilst these prophylactic vaccines give confidence to the antigenicity potential of oncogenic viral antigens, developments of the therapeutic branch are still underway.

#### 2.3.1. HPV

The Human Papillomaviruses (HPV) are a part of *Papovaviridae* family, in which also includes polyomavirus and simian vacuolating virus. To date, it has been identified more than 200 HPVs serotypes with the involvement of high-risk HPVs in the establishment and progression of cervical and other anogenital cancers, along with squamous cell H&N cancers [161]. Of the 12 HPV types (HPV16, 18, 31, 33, 35, 39, 45, 51, 52, 56, 58, and 59) classed under group 1 carcinogens by the International Agency for Research on Cancer (IARC), HPV16 and 18 are shown to display the highest carcinogenic capacity in humans [162]. On one hand, prophylactic HPV vaccines, such as Cervarix, Gardasil, and Gardasil 9, offer protection against a subset of cancer types. On the other, there is an urgent need for the development of a therapeutic cancer vaccine capable of targeting pre-existing, established HPV-associated cancers.

The early genes E6 and E7 are implicated in cellular oncogenesis. The most well-known oncogenic pathways include E6 and E7 interaction with two different tumour suppressor proteins, p53 and retinoblastoma protein (pRb) respectively [163]. The HPV E6 oncogene ubiquitinates p53 with the assistance of E6-associated protein (E6AP/UBE3A). By acting in a complex consisting of E6, E6AP and p53, the heterotrimeric complex is able to mediate ubiquitination of p53. The ubiquitination of p53 leads to the degradation of the protein and inhibition of apoptosis. Similarly, E7 also ubiquitinates pRb. pRb works in a complex with the E2F transcriptional network, serving as a checkpoint for cells between the G1 and S phases. The binding and degradation of pRb via E7 results in the release of these E2F transcription factors, with gene products such as cyclin E, cyclin A and p16^INK4A^. Collectively the expression of these genes contributes to the unrestricted transition of premature cells to the S phase, resulting in DNA synthesis and cell proliferation [161,164]. Besides the roles of E6 and E7 in the evasion of growth suppressors, their binding interactions and presence in other cellular pathways result in the two oncoproteins heavily involved across other cancer hallmarks including immortalization, sustained proliferation, induction of angiogenesis and also the activation of invasion and metastasis [163,164]. Given its widespread influences in promoting cell malignancy and foreign origin, both the E6 and E7 antigens are identified as key tumour-specific targets for the development of oncogenic viral antigen-based vaccines.

In an early Phase I clinical trials by Bagarazzi et al., a mixture of two plasmid DNA vaccines expressing the HPV-16 and HPV-18 E6/E7 oncogenes respectively (VGX-3100) was delivered by intramuscular electroporation (EP) to 18 grade 2/3 cervical intraepithelial neoplasia HPV16 or 18^+^ patients and reported that 100% of individuals displayed antigen-specific humoral immune responses to at least two of the vaccine antigens utilized, followed by 94% (17/18) and 56% (10/18) of patients responding positively to three and all of the cancer vaccine antigens, respectively [48]. Seventy-eight percent of patients developed HPV-specific cytotoxic CD8^+^ T-cells upon vaccination, of which 11 of 14 responders exhibited a persistent memory response measured at 24 after vaccination. Subsequently, VGX-3100 Phase II trials with HPV 16/HPV 18 grade 2 or 3 positive cervical intraepithelial neoplasia patients, showed a significant increase in signs of histopathological regression in vaccinated patients versus placebo from 30.6% (11/36) to 49.5% (53/107) (*p* = 0.034) [49]. Currently, the VGX-3100 vaccine is being trialled in an active Phase III study (REVEAL 1, NCT03185013) with cervical intraepithelial neoplasia grade 2/3 patients with an anticipated completion date in April 2021.

In a Phase III clinical trials, the direct injection of MVA vaccinia virus expressing the bovine papilloma virus E2 gene (MVA E2) in the uterus, urethra, vulva, or anus of 1356 patients with grade 1, 2, or 3 cervical intraepithelial neoplasia or condyloma lesions resulted in complete regression in 94.82% (825/870) and 73.33% (220/300) of female patients with low-grade and high-grade lesions respectively, alongside 100% (180/180) male patients with condyloma lesions in either the urethra or anus [50]. In the MVA E2-treated group, 5 females of 141 (3.54%) with high-grade lesions and none of the treated males experienced disease recurrence within two years after treatment. Although, all patients treated with conventional methods eliminated their lesions by 14 weeks after treatment, 89.36% (126/141) of the females and 100% (26/26) of the males in this group showed the reappearance of the same initial lesions after two years.

Another vaccine that has made progressed along the clinical development pipeline is a synthetic HPV16 E6 and E7 long peptides (25–35 mer) consisting of a pool of nine E6 and four E7 peptides overlapping 10–14 amino-acid sections (HPV16-SLP vaccine) in Montanide ISA-51 [51]. The Phase II study enrolled six patients afflicted by HPV16+ cervical carcinoma and resulted in the generation of long-lasting (up to 12 months) specific CD4^+^ and CD8^+^ T-cell responses against E6 (six in six patients) and E7 (five in six patients) antigens. A Phase II study by the same authors of HPV16-SLP-vaccine on HPV16-induced gynaecological carcinoma reported that unfortunately, none of the evaluable vaccinated patients displayed tumour regression associated with the vaccine and 19 patients succumbed to progressive disease (median OS = 12.6 months) [52].

Besides the progress observed in gynaecological carcinomas, vaccines that target other types of HPV-associated cancers are in development. In a recent Phase Ib/II study by Aggarwal et al., MEDI0457, a DNA plasmid vaccine expressing HPV-16 and 18 E6/E7 oncogenes supplemented with recombinant IL-12 plasmid was given to 21 HPV-associated H&N cancer patients resulting in 18 out of 21 individuals mounting a HPV-specific T-cell response detectable up to one-year after vaccination [53]. In four out of five responders, the vaccination regimen not only induced HPV-specific CD8^+^ T-cells but also shifted the ratio of CD8^+^/FoxP3^+^ T-cells (forkhead box P3 expression highlights immunosuppressive T-cells) and increased the numbers of perforin+ lesion infiltrates in all five patients. Of note, one patient who developed progressive disease was treated with anti-PD-1 resulting in a complete and durable response.

Our laboratory has recently finished a Phase I dose escalation clinical trials of the AMV002 vaccine, a DNA vaccine consisting of a mixture of NTC8485-O-UE6E7 and NTC8485-O-s-E6E7 plasmids that express a codon optimized recombinant HPV16 E6E7 fusion protein with a single ubiquitin sequence repeat (-O-UE6E7) or a murine IgK secretory sequence (O-s-E6E7). This DNA vaccine was trialled in conventionally treated HPV16^+^ Oropharyngeal Squamous cell carcinoma patients (OPSCC) with no evidence of recurrent and/or metastatic disease [54]. AMV002 was well tolerated and elevated specific T-cell immune responses to E6- and/or E7 antigens in 10 of the 12 treated patients (83.3%) with an observed four-fold increase in E6/E7 antibody titers in one out of four patients in the highest dose cohort (4 mg). Given the favourable responses observed within the Phase I trials, AMV002 is evaluated in a second Phase I trial in combination with an anti-PDL1 ICI in recurrent and/or metastatic OPSCC (ACTRN12620000406909).

#### 2.3.2. EBV

The Epstein-Barr Virus (EBV) is a member of the herpesvirus family along with human herpesvirus 8. Spread through salivary contact, EBV is common within adults (>90 % of the world’s population) and has been observed to increase the risk of cancer, such as Burkitt’s and Hodgkin lymphomas, diffuse large B-cell lymphoma, undifferentiated nasopharyngeal cancer, gastric adenocarcinoma and leiomyosarcoma [165]. Primary infection of EBV occurs via the oropharyngeal epithelium and B cells are affected when the gp350/220 glycoprotein is attached to CD21 molecule on the surface of B cells. In complex post-attachment events, crosslinked CD21 constitutes an activating signal for EBV binding and downstream processes which result in the delivery of the viral genome into the nucleus. The EBV viral genome is then circularized in the nucleus, resulting in the expression of its products, including the subset of EBNA proteins and the two latent membrane proteins (LMPs) [166,167]. Common antigens targeted in vaccines include the latent membrane proteins (LMP1 and LMP2) and the EBV nuclear antigen 1 (ENBA1). LMP1, via association with the tumour necrosis factor receptor-associated factors (TRAFs), mimics CD40 as a constitutively active receptor to induce cellular growth. Furthermore, signalling via TRAF and Tumour necrosis factor receptor type 1-associated DEATH domain proteins (TRADD) result in the activation of transcription factor, nuclear factor κB (NF- κB), elevating B-cell lymphoma 2 (BCL2) levels, resulting in the inhibition of apoptosis [168]. The LMP-2A protein has also been shown to activate the AKT pathway through the induction of phosphoinositide 3-kinase (PI3K), which contributes to apoptosis inhibition [169]. EBNA1, not only is in charge of the replication and maintenance of the EBV genome, but is associated with cellular transformation, through a yet unidentified mechanism [169,170]. As a result, tumour-specific antigen vaccine designs associated with EBV have historically been focused on the antigens listed. Therapeutic vaccines recorded in clinical development include MVA-EL and an adenovirus integrated dendritic cell vaccine in phase II trials. Two Phase I clinical trials conducted in Hong Kong [55] and the United Kingdom [56] with MVA-EL vaccinia vaccine encoding the full-length LMP2 and CD4^+^ T-Cell epitopes of EBNA1 with EBV-positive nasopharyngeal carcinoma patients reported that 23 out of 32 patients developed LMP2/EBNA1 specific CD8^+^ and CD4^+^ T-cell immunity (Hui et al., eight in 14 patients; Taylor et al., 15 in 18 patients). Currently, MVA-EL is being tested in a Phase Ib/II clinical study (NCT01094405).

Reported in 2012, patient autologous DCs transduced with an adenovirus encoding a truncated LMP1 (ΔLMP1) and full-length LMP2 (Ad-ΔLMP1-LMP2) was trialled in a Phase II study with metastatic EBV-positive nasopharyngeal carcinoma (NPC) [57]. However, neither LMP1/LMP2 nor adenovirus-specific T-cell responses were observed in treated patients, with only two individuals (25%) displaying immune responses to EBNA1 protein. The PFS of the vaccinated cohort was 1.92 months and the OS was 6.0 months, with positive OS correlating to lower EBV-DNA loads in peripheral blood. Of the 12 patients, three (25%) showed favourable clinical responses/outcomes, with one patient exhibiting partial responses to the vaccine for 7.5 months and the other two stable disease for 6.5 and 7.5 months, respectively.

#### 2.3.3. Hepatitis B and Hepatitis C

Hepatitis B and Hepatitis C (HBV, HCV) are a part of group of hepatovirus that affects the liver. In Hepatitis B, virion DNA is morphed into covalently closed circular DNA (cccDNA), generating a minichromosome that aids viral mRNA synthesis. The cccDNA serve as a template for six viral RNAs that encodes for seven viral proteins (HBV Core, HBV Polymerase, HBV preCore, HBV Surface proteins (L, M, and S)) [171]. In Hepatitis C, the polyproteins encoded from the RNA genome of the virus are co/post-translationally processed into a subset of >10 structural and non-structural viral proteins (Core, E1, E2/p7, NS2, NS3, NS4A, NS4B, NS5A and NS5B) [172]. Infection with HBV/HCV may be transient or chronic, but in many instances, chronic infection results in cirrhosis (impaired, scarred liver tissue) and in some cases, hepatocellular carcinoma (HCC), with 80% of HCC incidences suggested to be associated with HBV/HCV infection [173]. Whilst these viral proteins are implicated in a multitude of biological/physiological functions, some of the viral proteins listed above are associated with hepatocarcinogenesis and HCC. Whilst the list is not exhaustive, key proteins typically mentioned includes the HBx protein, HBV polymerase and hepatitis B surface proteins for Hepatitis B and proteins such as the HCV core, NS3, NS5A and NS5B represents the suite of hepatitis C proteins [171,172].

A Phase II study with a Hepatitis C vaccine based on the HCV core antigen and supplemented with 31 additional peptides derived from 15 unique tumour-associated antigens with 42 HCV-positive advanced HCC patients resulted in vaccine-specific T-cell responses in 19 out of 36 evaluable patients and reduction in serum concentrations of α- fetoprotein (AFP) and des-γ-carboxy prothrombin (DCP) in nine out of 33 treated patients [58]. Median OS for the 42 patients was 184 days, of which patients who showed decreased either AFP or DCP levels reported longer survival times (*n* = 13, median OS = 286 days) than non-responders (*n* = 26, median OS = 180 days) (*p* = 0.01).

## 3. Vaccine Delivery Systems

**Figure 2:**
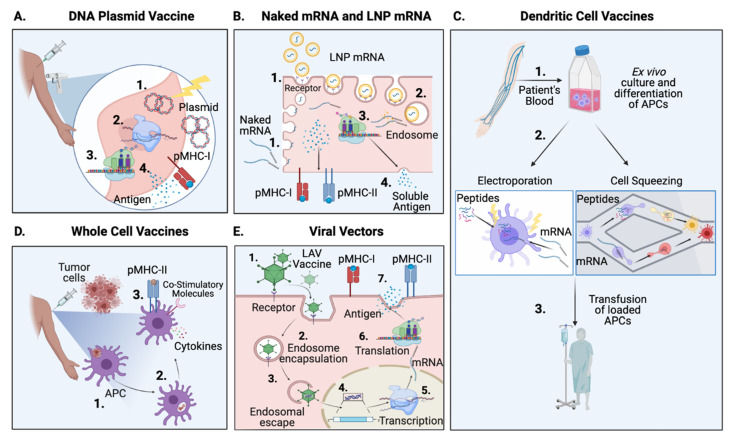
**Cancer vaccine delivery methods**. Various delivery strategies used to expose patients to immunogenic tumour antigens. (**A**) **DNA Plasmid Vaccine.** (1) DNA plasmids encoding cancer antigens are injected intramuscularly or pushed into myocytes using electrical pulses from a gene gun. (2) The host cells transcribe and (3) translate the given tumour antigens. (4) Tumour antigens peptides will be presented to immune cells on MHC-I molecules or alternatively secreted and then taken by APCs. (**B**) **Naked mRNA and Lipid Nanoparticle (LNP) mRNA.** (1) LNP mRNA is delivered to host cells via systemic or intramuscular injection and uptaken by the cells through specific ligand/receptor interactions. Naked mRNA is administered via intra-lymph node or intra-muscular injection. (2) Lower endosomal pH triggers LNPs to release mRNA cargo in the cytosol of the cell. (3) mRNAs are translated by the cell ribosomal machinery. (4) Tumour antigen peptides will be presented to immune cells as pMHC-I and/or pMHC-II (only on APCs) complexes. (**C**) **Dendritic Cell Vaccines.** (1) Ex vivo differentiation of patient’s blood cells into APCs. (2) APCs will be loaded with the tumour antigens of interest using either electroporation, where electrical pulses make cells temporarily permeable, or cell squeezing, where a microfluidic flow temporarily deforms the cell plasma membrane to create pores enabling the passage of therapy into cells. (3) Modified cells can then be transfused back into the donor patient. (**D**) **Whole Cell Vaccines.** (1-2) Subcutaneous injected allogenic or autologous tumour cells will be uptaken and processed by APCs. (3) Activated APCs secreting cytokines and expressing co-stimulatory molecules, will mediate the recognition of pMHC-II by T-cells (**E**) **Viral Vectors.** (1-3) Injected viral particles, enter the cell by receptor mediated endocytosis and released into the cytosol. (4) In the case of DNA life attenuated viruses, viral genome containing the encoded tumour antigens is transferred into the host nucleus and (5) transcribed into mRNA. (6) The therapeutic mRNA is translated in the cytosol to protein(s)/peptide(s). (7) mRNA products will be secreted either/or presented by MHC complexes on the surface of the cell. (Created in BioRender.com).

Advances in defining TAAs, neoantigens and oncolytic viral antigens have contributed to a rich source of options to treat cancer. The correct delivery of therapies is as important as the antigen selection, because if the proper immune response is not invoked, immune tolerance rather than activation of the immune system occurs [174]. Therefore, another aspect of cancer vaccine development is to define the means by which tumour immunogens are given to patients and presented to the immune system (Figure 2).

### 3.1. Peptide Vaccines

Peptides are short subunits of proteins resulting from cell mediated degradation processes and are presented on the cell surface via MHC for T-cell recognition. Given that they can be easily synthesized from known tumour antigen data, peptide-based vaccines have been and are actively researched upon as seen in previous sections of this review. Mechanistically, endogenous cell peptides (8–10 amino acid residues in length) are presented to cytotoxic CD8^+^ T-cells by MHC-I while longer peptides (13–25 amino acid residues) are presented to CD4^+^ T-cells by MHC-II. The function of MHC-I to CD8^+^ T-cell interactions are to primarily assess whether infection or tumorigenesis has occurred on a cell-to-cell basis. APCs, such as DCs, macrophages, or antibody producing cells such as B cells, present extracellular antigenic peptides on MHC-II, interacting with CD4^+^ helper T-cells. Activated CD4^+^ T-cells directly assist CD8^+^ T-cells by secreting IL-2 and maintain pro-inflammatory DCs. Indirectly, activated CD4^+^ T-cells secrete proinflammatory cytokines such as TNF-α and interferon gamma (IFN-γ) whilst assisting with B cell maturation. Environmental sampling of extracellular antigens by DCs, with subsequent cross presentation of antigen on MHC-I to CD8^+^ T-cells is another important mechanism by which cytotoxic CD8^+^ T-cell responses are generated against distant tumours [175,176]. The manner in which peptides are presented to elicit successful immune responses is crucial. When peptides are presented to T-cells in the absence of the requisite co-stimulatory signals, it results in the improper activation of immune cells, leading to tolerance and dysfunctional cell states, as opposed to a strong immune response [177]. In contrast to viral, cellular, and nucleic acid approaches discussed herein, peptides are not immunogenic themselves and require the presence of an adjuvant to initiate pro-inflammatory and co-stimulatory signals to attain favourable CD4^+^ and CD8^+^ T-cell responses. Owing to their ability to be degraded easily, peptide vaccine technology development is challenging, but progress in ex vivo DCs pulsing or nanoparticle delivery platforms will be covered in more detail in the sections ahead.

### 3.2. Nucleic Acid Vaccines (DNA & mRNA)

Nucleic acid vaccines comprise of DNA or mRNA have been gaining increased prominence in the fields of virology and oncology. Recent data have shown the safety and utility of mRNA-based vaccines for Covid-19 in humans [178,179]. Both, DNA and mRNA vaccines, ultimately induced host cells to express antigenic proteins or peptides which may trigger antigen-specific immune responses. The use of nucleic acid vaccines has the added advantage of being a self-adjuvating due to their ability to activate inflammatory pathways through interactions with TLR- 7 & -9 [180,181]. Both mRNA and DNA nucleic acid production methods can be scaled up, purified easily, and are good manufacturing practice (GMP) compliant, allowing for efficient commercial production [9,182]. There are two methods by which nucleic acid therapies can be delivered, either directly as naked DNA and mRNA or indirectly through delivery platforms such as a lipid nanoparticle (LNP) or virus like particles.

#### 3.2.1. Naked DNA Vaccines

Based on bacterial plasmids, DNA vaccines encode antigens under strong eukaryotic promoters, often of viral origin, which when delivered to cells will first be transcribed to mRNA, are subsequently translated into protein which may later be processed by APCs to induce both CD4^+^ and CD8^+^ T-cell mediated immune responses [183]. The indirect DNA delivery route requires intramuscular or intradermal delivery of naked DNA into myocytes through the use of a gene gun, nano particles, or microneedles. In contrast, direct delivery of DNA vaccines involves the ex vivo transfection of autologous APCs and subsequent transfusion of those cells back into the host’s body. Once within the cytosol of host cells, the DNA plasmid produces encoded antigens that can be displayed on the surface of the cell loaded in MHC-I molecules, or when transfected cells die and are processed by APCs, peptides displayed on MHC-II, resulting in CD8^+^ and CD4^+^ T-cell mediated immunity. Alternatively, soluble antigenic proteins are shed from host cells later picked up by APCs for presentation to T-cells [184].

A DNA-based vaccine to treat human papilloma virus 16/18 cervical high grade squamous intra epithelial lesion (HPV16/18 HSIL) represents the closest progression to an FDA approved DNA vaccine at present. Intramuscular injection of VGX-3100 plasmid (encoding HPV 16/18 E6 and E7 antigens) has completed Phase IIb and is currently in Phase III trials as discussed previously in Section 2.3. HPV (Figure 2A) [49].

#### 3.2.2. Messenger RNA Vaccines, Naked mRNA and DC-Delivery

Messenger RNA is a downstream product of DNA, and its function is to translate instructions from genomic DNA into functional protein products that are used by the host organism. The potential for mRNA as a therapeutic, was discovered when Wolff et al. observed the production of proteins after intramuscular injection of naked mRNA into mice [185]. Hoerr et al. later extended this work and demonstrated that intradermally administered naked or liposome encapsulated mRNAs led to local production of the antigen beta-galactosidase (β-gal) which induced the formation of β-gal-specific cytotoxic T-cells and antibodies (Figure 2B) [186].

In 2009, the ex-vivo co-electroporation of human DCs with a combination of immunostimulatory mRNAs encoding CD40 ligand, CD70 and a constitutively active TLR- 4 (TriMix DC) plus mRNAs encoding melanoma antigens MAGE-A3, MAGE-C2, gp100 and tyrosinase (TriMixDC-Mel), induced DC maturation and generated antigen-specific T-cells for the tumour associated antigens tested ex vivo [187]. In the same study, three patients with recurrent stage III or IV melanoma received four biweekly infusions of transduced DCs. In contrast to unvaccinated patients, a robust antigen specific CD8^+^ T-cells was generated by Tri-Mix DC therapy. In a separate experiment, the *in vitro* comparison of pulsed TriMix-DC or TriMix-Ipi with the immunodominant MelanA-A2 peptide or MelanA mRNA, resulted in similar induction profiles of MelanA-specific CD8^+^ T-cells, implying that both were effective at eliciting immunity [187]. Van Lint et al. extended these findings and demonstrated in multiple mouse models that the intra lymph node injection of TriMix mRNA in conjunction with antigen coding mRNA’s (tyrosinase-related protein-2 (Trp-2), Wilms’ Tumour gene 1 (WT1) or tyrosinase), induced efficient DC maturation and antigen-specific CD4^+^ and CD8^+^ T-cells as efficiently as transference of ex vivo loaded DCs [188].

A two-stage phase II clinical trial of autologous TriMix-DC co-administered with ipilimumab was trialled in 39 patients with unresectable Stage III or IV Melanoma (NCT01302496) and resulted 51% of the patients showing disease control at six months of which eight patients reached complete response, seven partial response and six showed stable disease [61]. In the five-year follow-up study, seven (*n* = 15) complete and one partial tumour responses were confirmed.

Recently, the delivery of mRNA has been also used to generate personalized melanoma vaccines (NCT02035956). As previously described in Section 2.2.3, this study elegantly demonstrated the first steps to the future of the intersection of novel in silico computational biology working symbiotically with molecular immune oncology to improve cancer patient survival outcomes.

While the use of naked mRNA injected therapies has been successful, the reliance on autologous DC pulsing and intranodal injections can be very expensive, uncomfortable to patient and requires specialized facilities. Recently, new *in vivo* delivery of therapies using particle-based approaches, such as lipid nano particle delivery of mRNAs to induce responses to prevent Covid-19 is one of the latest successes of this approach and can be adapted to deliver cancer therapy payloads to the body [178].

### 3.3. Particle Vaccines (LNP and VLP)

As previously discussed, peptides and nucleic acid therapies are susceptible to degradation. RNA therapies have benefitted from years of research to improve translation and resistance to degradation through modification of the 5′ cap and polyA tail, resulting in extended production within cells [189]. However, one limitation that both peptides and nucleic acid therapies need to circumvent is their uptake by cells. Pinocytosis is a typical mechanism by which therapies are known to enter the cell, but the efficiency is low [190]. Extensive research on delivery platforms have resulted in the development of lipid-based, polymeric, and virus like nanoparticles to support the efficient movement of cargo molecules across cell membranes.

#### 3.3.1. Lipid Nanoparticles in Cancer Vaccines Programs

Lipid nanoparticles (LNPs) are artificial spherical vesicles constructed using pH responsive lipids. Firstly, within an acidic environment (low pH), these LNPs are able to facilitate the therapeutic loading of biologics such as mRNA, DNA and peptides. When exposed to physiological pH they then become neutrally charged. The physical properties of these nanoparticles allow the efficient packaging of the cargo of interest, the uptake of LNPs by endocytosis with a low toxicity profile and finally release their cargo once a lower pH is encountered in the endosomes (Figure 2B) [191,192]. In addition to their recent role in mRNA therapy delivery, LNPs have been used to vaccinate against antigens encoded by small interfering RNA (siRNA) and small synthetic molecules or chemotherapeutic drugs such as paclitaxel [193].

##### Contemporary Uses of LNPs in Cancer Therapy

Delivery of mRNA methods have progressed from ex vivo loading of autologous DCs to systemic administration of neoantigen mRNA in LNPs. Recently, a Phase I clinical trial of a personalized neo-antigen vaccine called mRNA-4157 was delivered in LNPs (Moderna) to melanoma, bladder carcinoma, HPV-negative head & neck (H&N) squamous cell carcinoma, NSCLC, SCLC, and microsatellite colon cancer patients. There were four study groups: The first was therapy delivered as a monotherapy to resected tumours; the second group was a combination mRNA vaccine with Pembrolizumab (anti-PD-1) for patients with advanced and metastatic tumours; the third group were ICI naïve microsatellite colon cancer and HPV-negative HNSCC; and the fourth group resected melanoma patients. Fourteen out of 16 patients in the first group remained disease free at the end of the study (three melanoma, 10 non-small cell lung carcinoma, two small cell lung carcinoma, four microsatellite instability-high tumours). Of particular interest was that 50% of HPV-negative H&N squamous cell carcinoma that were ICI therapy naïve, 50% responded favourably to the mRNA-4157/pembrolizumab combination with a median PFS of 9.8 months (one complete response, four partial responses and four stable disease) in contrast to a median PFS of two months for 14.6% of patients enrolled in separate pembrolizumab monotherapy studies [47,194,195].

A Phase I study by Sahin et al. of an intravenously administered mix of four non-mutated shared melanoma mRNA TAAs (BNT-111: NY-ESO-1, MAGE-A3, tyrosinase and transmembrane phosphatase with tensin homology (TPTE)) delivered by LPX liposomes (FixVAC) with unresectable melanoma patients previously treated with ICIs reported potent and durable CD4^+^ and CD8^+^ antigen-specific T-cell responses in the presence or absence of co-administered anti PD-1 therapy [62]. Additionally, FixVAC had the ability to synergize with anti-PD-1 treatment reflected by the fact that two patients who received FixVAC after PD-1 therapy failure, underwent initial tumour regression before relapsing, to partially respond to a second round of anti-PD-1 therapy, an effect suggested to be the action of FixVAC induced PD-1^+^ T-memory cells that were sensitive to rechallenge with PD-1 therapy.

#### 3.3.2. Virus Like Particles

Unlike lipid nanoparticles, virus like particles (VLPs) are constructed from viral capsid and envelope proteins, assembling into structures with symmetry analogous to real viruses, but are non-infectious due to the lack of replicative components. Owing to their size, repetitive geometric structures, ability to manipulate their size to incorporate large payloads, and delivery of therapeutic nucleic acid formulations, VLPs are an emerging platform for cancer vaccines. VLPs are capable of delivering cancer therapies with inherent adjuvant activity, capable of migrating to areas such as lymph nodes, facilitating potent T-cells responses through DC mediated antigen presentation [196]. VLPs have been demonstrated to induce Th1 polarizing conditions that facilitate not only the production of pro-inflammatory cytokines but also the activation of antigen presenting cells. This in turn leads to the activation of CD4^+^, cytotoxic CD8^+^ T-cells, and B cell responses [196].

Gene delivering VLPs have been evaluated for treatment of melanoma, Breast, Pancreatic and Cervical cancers and Hepatocellular Carcinoma [197]. The VLPs has also been used to deliver adjuvants (Methylated/Unmethylated CpGs, QuilA, microcrystalline tyrosine) and small molecule chemotherapies (Gemcitabine), ICIs (anti-PD-1, anti-CTLA-4) or anti-regulatory T-cell therapies (anti-CD25 antibody- targets CD25 expressed on regulatory T-cells resulting in their depletion).

#### 3.3.3. Approved Virus Like Particle Therapies for Cancer

Prophylactic cervical and hepatocellular carcinoma cancers are approved for use in humans. By vaccinating against the causative viruses, HPV and Hepatitis B, a decline in their respective downstream cancers has been observed [198]. The prophylactic effect of these vaccines is key to eliminate these cancer types when given to populations prior viral exposure [199,200].

##### Human Papilloma Virus

Cervarix, Gardasil and Gardasil 9 are FDA approved prophylactic HPV vaccines, utilizing VLP technology that targets HPV-16/18 L1 proteins (Cervarix) and HPV-6, -11, -16, -18, -31, -33, -45, -52 and -58 L1 proteins (Gardasil-9), with >90% therapeutic efficacy observed in recipients [201,202]. The first generation vaccine prototype was created through vaccinia virus recombinants expressing Bovine papilloma virus L1 and L2 capsid proteins when transfected into mouse fibroblast cell lines [203]. Despite the differences between Cervarix and Gardasil, both vaccines produce potent immunogenicity and long-lasting neutralizing antibody titers. Vaccine efficacies for 4–6 months HPV 16/18 infection and disease endpoints reported by Harper et al. in women aged 15–26 years old for Gardasil and Cervarix were determined to be 96% and 94%, respectively, with a reduction to 85% and 91% in women aged above 25 years respectively [201]. Globally, Gardasil 9 has also demonstrated consistently high protection against HPV associated cervical cancers, with efficiencies at or above 90% in Africa, North America, Latin America and the Caribbean and efficiencies of 88% and 87% in Asia and Australia respectively [202].

##### Hepatocellular Carcinoma

HEPISLAV-B/ENGERIX-B, and more recently Sci-B-Vac, represent the most recently approved prophylactic VLP vaccines for the prevention of hepatocellular carcinoma by vaccinating against Hepatitis B. Both HEPISLAV-B and ENERGIX-B are VLPs utilizing Hepatitis B S antigen (adsorbed onto an alum backbone) with the difference that HEPISLAV-B incorporates the CpG adjuvant to induce immune responses. A comparison of superior seroprotection rates (anti-HBs ≥10 mIU/mL) of HEPISLAV-B and ENGERIX-B after their approved dosing schedules, resulted in higher rates of protection for the HEPISLAV-B cohort (91–100%) in contrast to the ENGERIX-B group (71–90%) with HEPISLAV-B protection also being more superior in poor vaccine responsive groups such as: older adults, diabetics and chronic kidney disease patients [204]. Sci-B-Vac, a tri-antigenic recombinant Hepatitis B vaccine, containing the small medium and large hepatitis B antigens, also generate potent immune responses. A recent phase IV study reported seroconvertion rates (anti-HBs ≥10 mIU/mL) for Sci-B-Vac recipients (healthy adults aged 20–40) in greater than 95% of patients [205].

### 3.4. Cellular Vaccines

One of the oldest and tenured forms of vaccine delivery systems are cellular vaccines. Within the contemporary setting, cellular vaccines include bacteria, tumours or more recently modified autologous DCs. The goal of the tumour and bacteria treatments are to act a source of antigen or immunostimulatory molecules to assist in the activation of immune responses. Modified autologous DCs, in contrast, are loaded with T-cell presenting antigens to initiate immune responses in the presence of relevant immunostimulatory signals.

#### 3.4.1. Dendritic Cells

Dendritic cells have been a logical choice to use in cancer therapy due to their ability to present antigens to both CD4^+^ and CD8^+^ T-cells and co-stimulate T-cells through molecules such as CD80 and CD86. While their use with nucleic acid therapies and tumour cells vaccines have been previously discussed with the only one DC-based vaccine approved for use in human prostate cancer (Sipuleucel-T, Section 2.1.2), other additional methods in which DCs have been modified in cancer vaccines are discussed below.

##### New Strategies to Load Antigens in APCs

Since their discovery in 1973, DCs have been identified as key antigen-presenting cells with great potential in vaccine development efforts. DCs are critical in immunosurveillance, where they aid in detection of malignancies. The basic principles to develop generic DC vaccine are as follows. Apheresis is undertaken in patients where autologous peripheral blood mononuclear cells (PBMCs) are obtained. Through processes of cell enrichment and various cell selection methods, such as fluorescence-activated cell sorting (FACS), naturally circulating DCs and or monocytes are isolated. As monocytes are undifferentiated forms of leukocytes, co-stimulation is required for the differentiation of monocytes into monocyte-derived dendritic cells. Maturation factors are subsequently supplemented to the two populations of DCs, where it is expected that mature cells are then capable of expressing major histocompatibility complexes I and II, along with co-stimulatory molecules. Once the cells are fully matured, the process of antigen loading occurs, where DCs are loaded with cancer tumour antigens. The mature antigens carried by DCs are then delivered to patients as a cellular vaccine [206].

However, throughout the manufacturing pipeline, differences arise from cell culture and maturation protocols, choice in DC populations, tumour antigen selection, antigen loading techniques and the route of administration [206,207,208]. These attempts of optimisations to DC vaccines are seen to be integral towards the development of a successful vaccine. Hence, in this section on vaccine delivery systems, recent novel and contemporary strategies employed for the loading of antigens are discussed as follows.

A 2013 study by the Jensen group at the Massachusetts Institute of Technology demonstrated the potential of a mechanical vector-free method for the introduction of materials into DC cells [209]. The technology called Cell Squeeze® infuses molecules of interest into recipient’s cell cytosols, by squeezing them through pores in a flow through cell, temporarily disrupting cell membrane integrity. During this time, payload molecules, in suspension with the cells, are free to migrate through the disrupted cell membranes, remaining inside as these gaps seal quickly after squeezing (Figure 2C). Recently, Squeeze® technology was used ex vivo to demonstrated that different antigens (synthetic long peptides for Cytomegalovirus (CMV) and HPV16^+^ tumours, neoantigens and M1 influenza mRNA) delivered to human PBMCS resulted in robust antigen-specific CD8^+^ T-cell responses highlighting the potential to leverage their platform across a wide variety of diseases [210]. In a separate study, the Cell Squeeze® platform was used to demonstrate that murine or human red blood cells could be converted into activating antigen carriers (AACs). In mice bearing selected tumour types, AAC’s carrying TAA synthetic long peptides capable of interacting with and activating macrophages and DCs without modifying CD47 expression (a multifunctional cell surface protein that mediates inhibition of phagocytosis, T-cell activation and is a regulator of inflammation) resulted in antigen-specific CD8^+^ T-cell and mediated anti-tumour responses measured by the reduction of tumour size [211]. Separately, human AAC’s loaded with peptides and adjuvants were demonstrated to promote, upon engulfment, monocyte derived dendritic cell (MODC) maturation, *in vitro* [211]. Next, a combination of cytosolic delivery with Cell Squeeze® technology of HPV E6/E7 antigens in combination with IL-2 variants (IL-2v), anti-PD-1 and anti-fibroblast activation protein (FAP) which collectively favours natural killer cells and CD8^+^ T-cell responses, resulted in significantly improved survival compared to monotherapy groups in a murine Human Papilloma Virus tumour model (TC-1 cells) [212]. Expansion of intra-tumoral antigen-specific CD8^+^ T-cells alongside enhanced TNF-α and IFN-γ production were responsible of the vaccine antitumour response. This work has been extended to a phase I dose escalation and expansion clinical trials (NCT04084951) that combines administration of HPV 16 E6/E7 Antigens loaded onto patient’s autologous PBMCs with or without the combination of atezolizumab (a monoclonal anti PD-L1 antibody), in HLA-A*02^+^ patients with HPV16^+^ recurrent locally advanced or metastatic solid.

#### 3.4.2. Whole Cell Vaccines

Besides DCs, another popular form of cellular vaccines pursued in research are whole cell vaccines. Either as autologous patient derived tumour cells or as allogenic cells derived from tumour cells, both forms of whole tumour cell vaccines have been investigated for clinical use in patients. In comparison to DCs, the use of whole tumour cells in the vaccines has the advantage of antigen accessibility. It is postulated that the full range of immunogens and antigens of interest can be administrated when the whole tumour cells are utilized as vaccine vectors, where characterization and identification events can be bypassed as part of vaccine design [213]. However, due to poor standardization of cytokine and chemokine concentrations to preserve whole tumour cells viable, advances have been made to develop genetically modified cells that contain costimulatory molecules required to induce desired immunity.

To date, GVAX vaccines are the most extensively studied whole cell vaccines. These vaccines are constructed with genetically modified whole tumour cells expressing GM-CSF (Figure 2D). Since its first use, GM-CSF was seen to be one of the best choices for a potent immunostimulatory cytokine [214]. At the time, it showed to promote the recruitment of APCs to the site of administration and to promote uptake of cancer tumour cells as part of cross-presentation. The cumulative effects of GM-CSF resulted in potent antitumour responses. Early preclinical trials in murine models displayed promising results, however, this was not reflected in studies with humans. Two Phase III (VITAL-1 and VITAL-2) clinical trials of prostate GVAX conducted in castration-resistant prostate cancer patients were ultimately terminated due to lack of efficacy [64,65,66].

Despite the lack of therapeutic effect observed in GVAX vaccines, other whole cell vaccines over the years have been investigated. Melacine was administered in stage IV melanoma patients, CancerVax was an allogeneic melanoma vaccine and OncoVax autologous cellular vaccines that were administered with Bacillus Calmette Guerin (BCG) in stage IIIA/IV melanoma and stage II colon cancer, respectively. Firstly, Melacine was a whole cell vaccine based on 2 melanoma cell lines, administered with the detox adjuvant [67]. In a study conducted by Southwest Oncology Group 9035 revealed that between vaccinated and control cohorts of Stage IIA melanoma patients, there were no statistical difference in DFS. However, the trial reported that subgroups of patients with cross reactivity with HLA-I antigens including HLA-A*022, HLA-A*028, HLA-B*044, HLA-B*045, and HLA-C*03 (predominantly HLA-A*022 and HLA-C*03) displayed superior clinical outcomes (5-years DFS 77% HLA-A2 and HLA-C3 positive patients, compared to 64% negative). On the other hand, Canvaxin (CancerVax), was an allogenic melanoma vaccine that was derived from 3 melanoma cell lines, administered intradermally with BCG. Whilst Phase II studies revealed a significant improvement in median OS and five-year OS in vaccinated patients vs. unvaccinated (56.4 vs. 31.9 months and 49% vs. 37%, respectively *p* = 0.0001), its Phase III and IV trials in both stage III and resectable stage IV patients were terminated when vaccine treated patients did not display improved OS [68,69]. Lastly, OncoVax was observed to increase DFS in stage II colon patients, supported by a 61% risk reduction for recurrence. Furthermore, increases in OS trends were reported as part of a secondary endpoint [70]. While the vaccine was approved for use in Switzerland, an ECOG study (E5238) reported that there were no significant clinical benefits for patients with stage II or III colon cancer [215]. Speculated reasons as to clinical failure of OncoVax has alluded to protocol consistency, quality control and patient selection [216].

#### 3.4.3. Bacteria

William Coley first used intra-tumoral injections of live Streptococcus pyogenes and later a combination of S. pyogenes and Serratia marcescens to induce tumour regression in 1891. Later mouse experiments using live BCG as an immunostimulant increased resistance to tumour implantation [217]. In 1976, these discoveries resulted in the use of BCG to successfully treat bladder cancer patients [218]. More recently, 64 metastatic melanoma patients received intravenous low dose (300 mg/m^2^) of cyclophosphamide (anti-inflammatory drug that inhibits regulatory T-cells) followed by an intradermal administration of a cancer vaccine consisting of irradiated autologous melanoma cells mixed with BCG. In the treated group, four complete and one partial disease regression with a median duration of 10 months were noted out of 40 treated patients [71]. Interestingly, the authors showed that delayed type hypersensitivity to the mechanically dissociated irradiated autologous melanoma cells correlated with anti-tumour responses. In a recent review, Wood et al. speculate that the transient inflammation associated with BCG related therapies is not sufficient to induce long-term durable cytotoxic T-cell responses and highlight the potential of *Listeria monocytogenes* bacterium as a candidate for cancer vaccine strategies [219].

*Listeria monocytogenes* is a gram-positive bacterium, capable of selectively infecting APCs, presenting tumour antigens to both MHC-I and -II pathways, resulting in the activation of CD8^+^ and CD4^+^ T-cells. An initial study by Le et al. in which 90 patients with metastatic pancreatic adenocarcinoma (97% of which had prior chemotherapy) were treated with low dose cyclophosphamide in combination with GVAX pancreas (Cy/GVAX) +/− CRS-207- a live attenuated *Listeria monocytogenes* expressing the pancreatic cancer antigen mesothelin. OS was superior when Cy/GVAX was co-administered with CRS-207 (OS 6.2 and 3.9 months respectively) [72]. Unfortunately, an expanded phase IIb study using the treatment did not recapitulate the initial study findings (OS of Cy/GVAX + CRS-207 and Cy/GVAX alone were 3.7 and 5.4 respectively) [73].

### 3.5. Delivery with Viral Vectors

The use of viruses in medicine is commonly known to be associated with vaccination against influenza, measles and varicella. The use of attenuated or replication deficient viruses has been gaining prominence in the treatment of cancer, owing to their ability to deliver selected antigenic payloads at the same time that strong innate and adaptive immune responses are induced (Figure 2E) [220]. One disadvantage of using viral vectors is that, for most viruses, after the first encounter with the immune system, a subsequently generated memory response will rapidly neutralize a second administration of the same virus, rendering repeat administrations of these types of vaccines useless over time [221]. To overcome this problem a heterologous prime-boost strategy delivering the same antigen over different viral vectors or combinations of virus and DNA vaccines can be used to induce the desired therapeutic outcomes.

A heterologous prime-boost strategy with vaccinia and fowlpox viruses has been used to treat prostate cancer using PROSTVAC-VF/Tricom [59]. Initially PSA on a vaccinia virus backbone was administered, with a later boost using PSA in fowlpox virus. While Phase II clinical trial data was encouraging in men with metastatic castration resistant prostate cancer (median OS for PROSTVAC versus empty vector was 26.2 versus 16.3 months, respectively) [59]. Phase III data did not meet acceptable endpoints and the trial was stopped [60]. Consequently, the PROSTVAC re-entered clinical trials, this time being co-administered with immune ICIs (NCT02933255 (recruiting 2021), (NCT02506114) (terminated low accrual)) in the hope of inducing more effective responses.

Vaccine-based immunotherapy regimen (VBIR) vaccines uses replication-defective chimpanzee adenovirus (ChAd68 serotype) expressing Prostate-Specific Membrane Antigen (PSMA) and Prostate Stem Cell Antigen (PSCA) to avoid pre-existing immunity, plus intramuscular electroporated DNA boost vaccination encoding PSA and PSMA [63]. VBIR approach in combination with tremelimumab (anti-CTLA-4) and RN888 (anti-PD-1) is currently in Phase I trials with prostatic cancer patients (NCT02616185).

### 3.6. Adjuvants

The relevance of co-administration of antigens and adjuvants to elicit therapeutic responses has been repetitively emphasised along this review. Adjuvants greatly vary in nature and modes of action [222]. In some instances, they function as delivery systems, assisting in the appropriately presentation of the selected antigens to APCs (ie., liposomes, viral particles), whilst in others, are immunostimulants that directly bolster the antigen-specific response by attracting the attention of the immune system to the delivery site (i.e., poly-IC) or modulating the type of response generated (i.e., IL-2). The adjuvants co-administered with cancer vaccines tested in clinical trials and named in this review are GM-CSF, Il-2, MIS416, poly-ICL, Montanide ISA-51, CHP and ISCOMATRIX and AS15. Briefly, GM-CSF, is an immunostimulatory cytokine that aids in the recruitment of DC to the site of vaccination [223]. It promotes DCs maturation and antigen presentation to heighten antigen-specific immune responses. The capacity of IL-2 to enhance the efficacy of vaccines is known since 1989 [224]. Since then, IL-2 and other cytokines have extensively been studied as vaccine adjuvants in both infectious and cancer scenarios. IL-2 promotes cellular immunity by stimulating the secretion of IFNγ by T cells and NKs and by skewing the T cell response towards the Th1 phenotype characterized by the activation of cytotoxic T cells and macrophages [225]. MIS416 is an adjuvant with bacterial origin developed by the Australian pharma Innate immunotherapies that targets APC via NOD2 and TLR9 pathways [226]. Poly-ICL derives from the combination of the synthetic double-stranded RNA poly-IC with poly-L-lysine in carboxymethylcellulose (CMC) which increases its resistance to nucleolytic hydrolysis. As an adjuvant, Poly-ICL strongly induces Th1 responses with the consequent secretion of type I and II interferons, as well as IL-12, TNF-α [227]. On the other hand, Montanide ISA-51 (Freund’s adjuvant), is a water-in-oil emulsion delivery adjuvant composed of a mineral oil and a mannide monooleate surfactant. Montanide ISA-51 is capable to enhance both humoral and cellular response by acting as a slow-release antigen depot which in turns promotes inflammation and lymphocyte recruitment [228]. CHP is a self-assembly polysaccharide pullulan with cholesteryl groups nanoparticle that delivers hydrophobic antigens to APCs at the same time that activates them [229]. ISCOMATRIX is a nanoparticle composed of a mixture of Quillaia saponaria extract, phospholipid and cholesterol that can deliver to APC up-to 50nm size payloads. ISCOMATRIX induces both Th1 and Th2 responses with the characteristic secretion of IFN-γ and IL-5 resulting in enhanced humoral and cytotoxic T cell responses [230]. Lastly, AS15 is a combinatory delivery and stimulatory adjuvating system based on MPL, QS-21 and CpG ODNs 7909. CpG ODNs 7909 is a synthetic 24-mer oligonucleotide containing 3 CpG motifs that activated DCs and B cells via TLR9 [231]. MPL derives from the lipid A, a component of *Salmonella minnesota* lipopolysaccharide (LPS) and promotes humoral and cellular immunity via TLR4 [232]. QS-21 naturally derives from saponin with various immunostimulatory activities [233]. Combinations of MPL and QS-21 are believed to act synergistically to support the induction of both humoral and cellular responses [234]. Overall, different adjuvants have different mechanisms of action to bolster antigen-specific immune responses and the theoretical/practical applications of adjuvants represent a broad and expanding field of research.

## 4. Discussion

Vaccines are one of the biggest successes of modern medicine. The World Health Organization (WHO) estimates that 4–5 million deaths and thousands of life-lasting disease sequelae are prevented each year with the currently running vaccination programs worldwide [235]. The latest and potentially most impactful examples are the Covid-19 vaccines. As recently as 19 April 2021, the Disease Control and Prevention (CDC) released to Cable News Network (CNN) the first public data about the impact that the Covid-19 vaccination campaign is having on the USA population revealing that from ~79 million fully vaccinated Americans, there were only 5800 Covid-19^+^ cases and 74 deaths [236]. Covid-19 vaccines are demonstrating to be objectively effective in real-life scenarios, not only being essential to prevent virus dissemination, but also to significantly ameliorate disease mobility and mortality.

Owing to their capacity to induce long-lasting protection, alleviate disease symptoms and reach large numbers within the population, the use of vaccines to elicit immunity against cancer is a logical approach. However, in contrast to the development of vaccines against foreign pathogens, achievements in the field of cancer vaccines have been modest with only one vaccine, Sipuleucel-T to treat prostate cancer, formally approved by the FDA.

Most of the clinical trials have focused on overexpressed TAAs (e.g., MUC-1, HER2/neu, p53 and hTERT) [8]. While most of these experimental approaches showed induction of vaccine-specific responses, these seem not to result in clinical benefits. The main issue with overexpressed TAAs is that in the end they are self-antigens and mechanisms of central and peripheral tolerance are in place to precisely avoid the generation of autoreactive B- and T-cells that strongly recognize these sequences [104]. Vaccines aiming to mount a response against these types of antigens need to overcome tolerance, goal attempted through the combination of strong adjuvants, co-stimulatory molecules and appropriate selection of new delivery formulations to amplify the immune stimulus leading to activation and expansion of self-antigen-reactive T-cells. To date, the cellular responses achieved with this approach have not resulted in the T-cell numbers or quality needed to be efficacious. Additionally, as overexpressed TAAs vaccines become stronger, the risks of autoimmune reactions also increase, drawing a fine line between potential benefits and tolerable side-effects. Fortunately, another type of self-antigen TAAs have been more successful. Normal differentiation antigens are not present in adult tissues, but their expression can be regained by the accumulation of aberrations leading to tumour development (e.g., gp100, PAP). In 2010, the FDA approved an APC-based vaccine (Sipuleucel-T) against PAP to treat metastatic castrate-resistant prostate cancer patients demonstrating that the underlying principles of a TAA-based vaccine are achievable [13]. However, the success of this first-of-the-kind PAP vaccine has not been followed by other normal differentiation antigens, highlighting the need for case-by-case testing. Both overexpressed and normal differentiation TAAs are subjected to different degrees of central tolerance. Conversely, cancer-germline/cancer testis antigens are restricted to immune privileged organs where these types of TAAs can avoid tolerization (e.g., MAGE-A3, NY-ESO-1) [119]. Clinical trials using MAGE-A3 and NY-ESO-1 proteins have failed to improve overall survival. However, the introduction of new technologies and the compilation of practical knowledge surrounding how to unlock tumour immune suppression is facilitating significant advances in the field of immunotherapy. A recent example is the reformulation of MAGE-A3, NY-ESO-1, Tyrosinase and TPTE antigens given to melanoma patients as mRNA delivered by LNPs resulting in three (*n* = 25) patients experiencing partial response, seven (*n* = 25) stable disease, and six (*n* = 17) patients that received anti-PD-1 developing partial response [62].

Neoantigens arise from de novo generated non-synonymous tumour mutations, and as such are “seen” by the immune system as foreign antigens avoiding tolerance [136]. By definition neoantigens are unique to each patient, and their abundance varies between cancer types [237]. Thus, the use of neoantigen in vaccines needs to be tailored to each patient. The wide adoption of high-throughput technologies has enabled such a personalized approach, with the first clinical studies starting to glimpse their therapeutic potential. Recently, two Phase I clinical trials of melanoma neoantigen-vaccines in the form of peptide or mRNA [43,44], resulted in both CD4^+^ and CD8^+^ vaccine-specific T-cell immunity, with a bias towards CD4^+^ T-cell responses, and clinical efficacy ranging from no recurrence in ~2 out of three of patients, to two (*n* = 5) relapsed patients favourably responding to post-vaccination anti-PD-1 treatment by the end of the study. These early promising results, encourage the immediate optimization of neoantigen vaccine development pipelines to reduce costs and complexities as well as to increase prediction accuracy to unleash the full potential of neoantigen cancer vaccines.

Oncogenic viral antigens are a strong source of foreign immunogens, which in some cases, are also drivers of cell transformation [160]. HPV and Hepatitis B prophylactic vaccines are a good example of the type of protective response viral antigens can confer by eliciting neutralizing antibodies that prevent the progression to chronic infection and subsequent virus-induced neoplasia. Unfortunately, once the virus has become established, these vaccines lack therapeutic effect and strategies that evoke cellular immunity are needed [199,200]. Multiple combinations and formulations expressing HPV E2 protein or E6 and E7 oncogenes, ranging from peptide vaccines to DNA modified plasmids, have been tested in pre-cancerous HPV^+^ intraepithelial neoplasia, cervical cancer and HPV^+^ H&N SCCs, where they have demonstrated good tolerability and the induction of vaccine-specific T-cell responses. However, some of the tested vaccines did not offered survival benefits and others are still in the process of being clinically evaluated as monotherapy or in combination with ICIs [49,54]. Some of these therapeutic vaccines prevent the reappearance of lesions in the mild forms of the HPV-derived disease [50]. Early clinical studies of cancer vaccine delivering EBV and Hepatitis C antigens respectively, have shown promising antigen-specific cellular immunity, which quality and strength would need to be assessed in randomized Phase III clinical trials [151,152,157].

Encouragingly, the numerous clinical attempts, while ultimately unsuccessful as treatments, have taught us invaluable lessons regarding strategies and formulations capable of delivering stronger responses than have been achieved previously. For example, we now know that for a cancer vaccine to mount a therapeutic response, the co-administration of multiple antigens alongside immune co-stimulatory molecules is likely to be necessary. A good example of this is the DC vaccine TriMix DC-Mel that combines the mRNA of both cancer-germline and normal differentiation melanoma antigens resulting in ~50% of patients developing complete response at five years post treatment [61]. The combination of “off-shelf” TAAs, patient-specific TAAs and patient’s neoantigens has also shown specific cellular response against all antigen types in a Phase I clinical trials with newly diagnosed glioblastoma patients [46]. The growing understanding of the key elements that inhibit or activates the anti-tumour response is revolutionising the field of immunotherapy. This has enabled the combination of antigen-based therapies with drugs, like ICIs, with the potential to target specific cellular responses and to eliminate both tumour and lymphocyte-mediated immune suppressive mechanisms. As such, many active cancer vaccine trials are exploring this avenue (e.g., SurVaxM + anti-PD-1; AMV002 + anti-PDL1; SQZ-PBMC-HPV + anti-PDL1). Results from Phase I and II studies of these types of combinations either attributed the positive effect to the ICI therapy or slightly lean towards a beneficial additive effect when vaccines are used alongside ICIs [33,47,62,194,195]. The truth is that most of these studies have been conducted under compassionate grounds with patients who have failed previous gold-standard treatments. Unfortunately, the extent to which these patient’s dysfunctional immune systems can respond is as-yet unknown. Huge progress has also been made with respect to delivery methods. Currently, a vast repertoire of systems ranging from LNPs and autologous DCs to genetically modified attenuated viruses, are available to systemically or locally evoke the desirable immune response to their payloads [238]. There is still much work that needs to be done to experimentally verify which antigen combinations delivered by which system in combination with which co-stimulatory drugs are able to break tolerance, revert immunosuppression, destroy tumours, and generate long-lasting memory responses which are the goals of efficacious cancer vaccines. As fast as the field evolves, it is logical to think that most of the elements to develop an efficacious vaccine are already at our reach, and that global efforts will help to identify through clinical data the winning combinations.

## Figures and Tables

**Table 1:** **Cancer vaccine Clinical Trials.** Selection of most relevant cancer vaccine clinical trials including characteristics and outcomes.

Antigen	Vaccine Name	Vaccine Type	Indication	Clinical Trial #	Clinical Outcome	Ref #
**MUC1**	Tecemotide/ L-BLP25	Peptide	Stage III non-small-cell lung cancer, completed chemoradiotherapy	NCT00409188 (Phase III)	Median OS: 25.6 months to 22.3 months (treatment to control)	[14]
	STn-KLH	Glycopeptide	Metastatic Breast Cancer	Unlisted (Phase III)	Median OS: 23.1 months to 22.3 months (treatment to control) Median PFS: 3.4 months to 3.0 months (treatment to control)	[15]
	PANVAC-V/F	Viral	Stage IV pancreatic cancer	NCT00088660 (Phase III)	Cancelled due to lack of clinical efficacy	[16,17,18]
**HER2/neu**	E75	Peptide	High risk node negative breast cancer	NCT00841399 NCT00584789 (Phase I/II)	5-year DFS: 89.7 % to 80.2 % (treatment to control)	[19]
	E75	Peptide	High risk node negative breast cancer	NCT01479244 (Phase III)	3-year Kaplan–Meier estimated DFS: 77.1 % to 77.5 % (treatment to control)	[20]
**p53**	SLP-p53^®^	Peptide	Epithelial ovarian cancer, with observed elevated levels of CA-125	Unlisted- Trial approved by Medical Ethical Committee of the University Medical Center Groningen (Phase II)	Stable Disease observed in 2/20 patients	[21]
	SLP-p53^®^	Peptide	Epithelial ovarian cancer, with observed elevated levels of CA-125	NCT00844506 (Phase II)	Stable Disease observed in 2/10 patients	[22]
	SLP-p53^®^	Peptide	Colorectal Cancer	ISRCTN43704292 (Phase I/II)	N/A	[23]
	SLP-p53^®^	Peptide	Platinum-resistant ovarian cancer	NTC01639885 (Phase I/II)	Partial Response observed in 2 patients and stable disease observed in 4 patients	[24]
	ALVAC	Cellular/Viral	Colorectal Cancer	Unlisted- Trial approved by local and national medical ethics/ biological safety committee and the Dutch Ministry of Health and Environment (Phase I)	N/A	[25]
	MVAp53	Viral	Recurrent epithelial ovarian/peritoneal/fallopian tube cancer	NCT02275039 (Phase I)	Median PFS: 3.0 months (treatment)	[26]
**hTERT**	GV1001	Peptide	Advanced / Metastatic Pancreatic Cancer	ISRCTN4382138 (Phase III)	Median OS: 6.4 months to 6.6 months to 4.5 months (control to concurrent treatment to sequential treatment) Median PFS: 7.89 months to 6.94 months to 8.36 months (control to concurrent treatment to sequential treatment)	[27]
	UVI	Peptide	Hormone-naïve prostate cancer	NCT01784913 (Phase I/IIa)	Stable disease observed in 17 of 21 treated patients	[28]
	VX-001	Peptide	Stage IV non-small cell lung cancer	NCT01935154 (Phase II)	Median TTF: 3.6 months to 3.5 months (treatment to control) Median OS: 14.3 months to 11.3 months (treatment to control)	[29]
**Survivin**	SurVaxM	Peptide	Recurring malignant glioma	NCT01250470 (Phase I)	Median OS: 86.6 weeks Median DFS: 17.6 weeks	[30]
	SurVaxM	Peptide	Newly diagnosed glioblastoma	NCT02455557 (Phase II)	Median PFS: 13.9 months	[31]
	SurVaxM	Peptide	Recurring malignant glioblastoma	NCT04013672 (Phase II)	Active Trial	N/A
	EMD640744	Peptide	Solid Mass tumours (metastatic or locally advanced)	NCT01012102 (Phase I)	Stable disease observed in 28% of treated patients	[32]
**Gp100**	MDX-1379	Peptide	Metastatic, unresectable Stage III/IV Melanoma	NCT00094653 (Phase III)	Median OS: 10.0 months to 6.4 months to 10.1 months (Treatment to monoclonal antibody monotherapy to vaccine monotherapy) Median PFS: 2.76 months to 2.86 months to 2.76 months (Treatment to monoclonal antibody monotherapy to vaccine monotherapy)	[33]
	gp100:209–217 (210V)	Peptide	Advanced Stage III cutaneous melanoma/IV melanoma	NCT00019682 (Phase III)	Median OS: 17.8 months to 11.1 months (treatment to IL-2 monotherapy) Median PFS: 2.2 months to 1.6 months (treatment to IL-2 monotherapy)	[34]
**PAP**	Sipuleucel-T (Provenge^®^)	Cellular- Dendritic Cell	Hormone-refractory prostate cancer	Unlisted- Trial approved by local institutional review boards at each study center and all patients signed institutional review board approved informed consent (Phase II)	N/A 38% of patients developed immune responses to PAP. Decline in PSA level by >50% was observed in 3 patients, and 25–49% in another 3	[35]
	Sipuleucel-T (Provenge^®^)	Cellular- Dendritic Cell	Metastatic, asymptomatic hormone-refractory prostate cancer	Unlisted- Trial approved by local institutional review boards at each study center and all patients signed institutional review board approved informed consent (Phase III)	Median OS: 25.9 months to 21.4 months (treatment to control) Median PFS: 11.7 months to 10.0 months (treatment to control)	[36]
	Sipuleucel-T (Provenge^®^)	Cellular- Dendritic Cell	Advanced prostate cancer	NCT00005947 NCT01133704 (Phase III)	Median OS: 23.2 months to 18.9 months (treatment to control) A 33% reduction in risk of death was observed for treated patients	[37]
	Sipuleucel-T (Provenge^®^)	Cellular- Dendritic Cell	Castration resistant prostate cancer	NCT00065442 (Phase III)	Median OS: 25.8 months to 21.7 months (treatment to control) A 22% reduction in risk of death was observed for treated patients	[38]
**MAGEA3**	recMAGE-A3	Protein	Stage IB, II, IIIA MAGE-A3-positive non-small cell lung cancer	NCT00480025 (Phase III)	Median DFS: 60.5 months to 57.9 months (treatment to control)	[39]
	recMAGE-A3	Protein	Stage IIIB/IIIC MAGE-A3-positive melanoma	NCT00796445 (Phase III)	Median DFS: 11.0 months to 11.2 months (treatment to control)	[40]
**NY-ESO-1**	CHP-NY-ESO-1	Peptide	Urothelial cancer, Prostate Cancer, Malignant solid tumours	UMIN000005246 UMIN000008006 (Phase I)	N/A	[41]
	NY-ESO-1/ iscomatrix	Peptide	Resected Stage IIc, IIIb, IIIc and IV melanoma	LUD2003-009 (Phase II)	Median DFS: 4.67 months to 5.79 months (treatment to control)	[42]
**Neoantigen**	Personalized Neoantigen Vaccine	Peptide	Stage IIB/C and IVM1a/b melanoma	NCT01970358 (Phase I)	4 of 6 treated patients had no disease recurrence at 2-years follow up. Other 2 patients experienced total regression post anti-PD-1 therapy	[43]
	IVAC Mutanome/ RBL001/002	mRNA	Stage IIIA-C/IV NY-ESO-1 and/or tyrosinase positive melanoma	NCT02035956 (Phase I)	8 of 13 treated patients had no disease recurrence at 1-2 years follow up. 2 out of 5 patients with recurrent disease showed objective response to vaccination with delayed relapse	[44]
	Personalized Neoantigen Vaccine	Peptide	Newly diagnosed (MGMT)-unmethylated glioblastoma	NCT02287428 (Phase I)	Median OS: 16.8 months Median PFS: 7.6 months	[45]
	APVAC2	Peptide (TAA + neoantigen)	Newly diagnosed glioblastoma	NCT02149225 (Phase I)	Median OS: 29.0 months Median PFS: 14.2 months	[46]
	mRNA-4157	mRNA	Melanoma Bladder carcinoma HPV-negative head & neck squamous cell carcinoma Non-small cell lung cancer Small cell lung cancer Microsatellite colon cancer	NCT03313778	Active Trial CPI-naïve HPV-negative HNSCC patient median PFS: 9.8 months	[47]
**HPV (E6/E7)**	VGX-3100	DNA	Cervical intraepithelial neoplasia grade 2/3	NCT00685412 (Phase I)	N/A	[48]
	VGX-3100	DNA	Cervical intraepithelial neoplasia grade 2/3	NCT01304524, EudraCT2012-001334-33 (Phase II)	Histopathological regression observed in 49.5% of treated patients to 30.6% in the control subgroup	[49]
	MVA E2	Viral	HPV intraepithelial lesions	Unlisted- Trial approved by Ethics and Scientific Committee of hospitals and corresponding health authorities from Estado de Mexico, (Phase III)	Complete regression observed in 94.82% (825/870) and 73.33% (220/300) of female patients with low-grade and high-grade lesions. Complete regression observed in 100% of male patients enrolled	[50]
	HPV16-SLP	Peptide	HPV16-positive cervical carcinoma	Unlisted- Trial approved by Medical Ethical Committee of the Leiden University Medical Center (Phase II)	N/A	[51]
	HPV16-SLP	Peptide	HPV16-induced advanced or recurrent gynecological carcinoma	Unlisted- Trial approved by Medical Ethical Committee of the Leiden University Medical Center (Phase II)	Median OS: 12.6 months	[52]
	MEDI0457	DNA	HPV associated head and neck squamous cell carcinoma	NCT02163057 (Phase Ib/II)	12-months DFS: 89.4% of treated patients	[53]
	AMV002	DNA	HPV-associated oropharyngeal squamous cell carcinoma	ACTRN12618000140257 (Phase I)	N/A	[54]
	SQZ-PBMC-HPV	Cellular- Whole Cell	HPV16+ Recurrent, Locally Advanced or Metastatic Solid Tumors	NCT04084951 (Phase I)	Active Trial- Recruiting	N/A
**EBV** **(LMP1/2)**	MVA-EL	Viral	Nasopharyngeal Carcinoma	NCT01256853, NCT01147991 (Phase I trials)	N/A	[55,56]
	Ad-ΔLMP1-LMP2 transduced DCs	Cellular—Dendritic Cell	Epstein–Barr virus (EBV)-positive nasopharyngeal carcinoma	Unlisted- Trial approved by Institutional Review Board of the National Cancer Centre, Singapore (Phase II)	Median OS: 6.0 months Median PFS: 1.92 months Of 3 out of 12 treated patients, 1 patient exhibited partial responses to the vaccine for 7.5 months. The other 2 patients maintained stable disease for 6.5 and 7.5 months	[57]
**HCV** **(HCV Core)**	C-35 peptide vaccine	Peptide	HCV-positive advanced hepatocellular carcinoma	UMIN000003520, UMIN000005634 (Phase II)	Median OS: 6.05 months	[58]
**PSA**	PROSTVAC-V/F-Tricom	Viral	Metastatic castration resistant prostate cancer	NCT00078585 (Phase II)	Median OS: 26.2 months to 16.3 months (treatment to control)	[59]
	PROSTVAC-V/F-Tricom	Viral	Metastatic castration resistant prostate cancer	NCT01322490 (Phase III)	Median OS: 34.4 months to 32.2 months to 34.3 months (PROSTVAC-VF monotherapy to PROSTVAC-VF + GM-CSF to control)	[60]
**Multiple Antigens**						
MAGE-A3, MAGE-C2, tyrosinase, gp100	TriMix-DC	mRNA	Stage III/IV Melanoma	NCT01302496 (Phase II)	Tumor response observed in 38% of treated patients, 8 complete and 7 partial responses were observed. 6 patients displayed stable disease. In 5-years follow-up, 7 complete and 1 partial response observed (*n* = 15) Median PFS: 6.21 months Median OS: 13.57 months	[61]
NY-ESO-1, MAGE-A3, Tyrosinase TPTE	BNT-111	mRNA	Advanced unresectable melanoma	NCT02410733 (Phase I)	Active Trial	[62]
PSA PSMA PSCA	VBIR	Viral	Prostate Cancer	NCT02616185 (Phase I)	Trial Completed as of 9 March 2021	[63]
**Undefined Antigens**	GVAX^®^	Cellular- Whole Cell	Asymptomatic prostate cancer	NCT00089856 (Phase III)	Trial terminated based on IDMC recommendation, with 30% chance of meeting primary endpoint of improving OS. OS reported post-study revealed median OS: 20.7 months to 21.7 months (treatment to standard care)	[64,65]
	GVAX^®^	Cellular- Whole Cell	Metastatic hormone refractory prostate cancer	NCT00133224 (Phase III)	Trial terminated following increased deaths in treatment arm to control	[65,66]
	Melacine	Cellular- Whole Cell	Resected primary cutaneous melanoma	Unlisted	5-years DFS: 77% for treated patients	[67]
	Canvaxin	Cellular- Whole Cell	Stage III Melanoma	Unlisted- Trial approved by UCLA/ JWCI–Saint John’s Health Center Institutional Review Boards (Phase II)	Median OS: 56.4 months to 31.9 months (treatment to control) 5-years OS: 49% to 37% (treatment to control)	[68]
	Canvaxin	Cellular- Whole Cell	Stage III/IV Melanoma	Unlisted (Phase III)	Study was terminated as a result of an interim analysis, concluding low probability of demonstrating significant improvement in survival	[69]
	OncoVax	Cellular- Whole Cell	Colon Cancer	Unlisted- Trial approved by participating hospital boards in the Netherlands, (Phase III)	61% risk reduction associated with longer recurrence-free period was observed in Stage II colon patients	[70]
	Unnamed Vaccine	Cellular- Whole Cell	Stage II/III Metastatic Melanoma	Unlisted (Phase I)	5 of 40 assessable, treated patients reported a median PFS of 10.0 months	[71]
	GVAX + CRS-207	Cellular- Whole Cell/Viral	Metastatic pancreatic adenocarcinoma	NCT01417000 (Phase II)	Median OS: 6.2 months to 3.9 months (CRS-207 co-administration with GVAX/Cyclophosphamide to GVAX/Cyclophosphamide monotherapy)	[72]
	GVAX + CRS-207	Cellular- Whole Cell/Viral	Metastatic pancreatic adenocarcinoma	NCT02004262 (Phase IIb)	Median OS: 3.7 months to 5.4 months to 4.6 months (CRS-207 co-administration with GVAX/Cyclophosphamide to GVAX/Cyclophosphamide monotherapy to control)	[73]
